# Multiobjective optimization to assess dengue control costs using a climate-dependent epidemiological model

**DOI:** 10.1038/s41598-023-36903-w

**Published:** 2023-06-24

**Authors:** Amália Soares Vieira de Vasconcelos, Josenildo Silva de Lima, Rodrigo Tomás Nogueira Cardoso

**Affiliations:** 1 Postgraduate Program in Mathematical and Computational Modeling (PPGMMC), Federal Center for Technological Education-CEFET-MG, Av. Amazonas, 7675, Nova Gameleira, Belo Horizonte, Minas Gerais 30510-000 Brazil; 2 Department of Mathematics, Federal Center for Technological Education-CEFET-MG, Av. Amazonas, 7675, Nova Gameleira, Belo Horizonte, Minas Gerais 30510-000 Brazil

**Keywords:** Applied mathematics, Computational models, Population dynamics, Viral infection, Differential equations, Nonlinear dynamics, Numerical simulations, Disease model, Health policy, Epidemiology

## Abstract

Arboviruses, diseases transmitted by arthropods, have become a significant challenge for public health managers. The World Health Organization highlights dengue as responsible for millions of infections worldwide annually. As there is no specific treatment for the disease and no free-of-charge vaccine for mass use in Brazil, the best option is the measures to combat the vector, the *Aedes aegypti* mosquito. Therefore, we proposed an epidemiological model dependent on temperature, precipitation, and humidity, considering symptomatic and asymptomatic dengue infections. Through computer simulations, we aimed to minimize the amount of insecticides and the social cost demanded to treat patients. We proposed a case study in which our model is fitted with real data from symptomatic dengue-infected humans in an epidemic year in a Brazilian city. Our multiobjective optimization model considers an additional control using larvicide, adulticide, and ultra-low volume spraying. The work’s main contribution is studying the monetary cost of the actions to combat the vector demand versus the hospital cost per confirmed infected, comparing approaches with and without additional control. Results showed that the additional vector control measures are cheaper than the hospital treatment without the vector control would be.

## Introduction

Dengue is the primary infection transmitted by the *Aedes aegypti* mosquito, registering more than one million cases in Brazil in 2022^1^. Therefore, there is an urgent need to develop and implement sustainable programs to prevent and control biological vectors, which are the transmitters of the causative agent of these diseases. Factors such as uncontrolled urban growth, climate change, and the lack of public awareness have aroused interest in studies in the area of mathematical epidemiology, especially to properly manage the financial resources destined for vector control and treatment of patients.

In this sense, many works have used mathematical models to analyze the behavior of the dengue vector. As explicit solutions are not always found, numerical methods are also part of the modeling. Thus, genetic algorithms are implemented to solve problems through a numerical approximation. Among the related works, we can mention^2–8^.

Given the above, it would be very relevant for a health manager to plan *Ae. aegypti* control actions for the coming years in the most effective way possible. For that, the decision-making process could be supported by information about the monetary value of the control versus the lower financial cost of treating patients infected with the dengue virus.

This work presents a novel epidemiological mathematical model for controlling the *Ae. aegypti* using climate variables. Data from humans infected with classic dengue, dengue with signs of alarm, and severe dengue were used to fit the model to represent the dynamics of the disease in an epidemic year in the city of Belo Horizonte, Brazil.

The aim is to study the effect of controlling the vector *Ae. aegypti*, based on actions already carried out by the Belo Horizonte City Hall. Additional control actions were proposed to reduce the number of infected humans, with larvicide and adulticide controls and ultra-low volume spraying, accounting for the cost of these operations through a genetic algorithm for multiobjective optimization.

The objective is to verify what would be the additional amount of control necessary to reduce the number of infected humans, taking into account the financial cost of both the control itself and the hospital treatment per infected person. In addition, also to reduce vector populations, the best time to start control actions is investigated. The results indicate that using the computational mathematical framework proposed in this work, in fact, may help in planning dengue control actions and reduce the number of infected and, therefore, the hospital cost.

## Materials and methods

### The dynamic system model

We propose a novel epidemiological mathematical model to represent the populations of the vector *Ae. aegypti* and human populations and the interactions between these populations for the spread of dengue. In this work, it is essential to consider the immature and adult populations of the vector for the application of control via larvicides and adulticides, respectively. Moreover, the asymptomatic infected human population should be included simultaneously due to its relevant role in dengue transmission dynamics since it can infect mosquitoes even without showing symptoms^9^.

There is a consensus in the literature that climate variables influence some of the entomological parameters of the model, so these parameters vary over time^3,4,10,11^. Therefore, another essential addition to the model that makes it more realistic is the dependence of some of its parameters on three climatic variables: temperature, precipitation, and humidity. The proposed model can represent any generic place by adjusting its parameters according to any city’s climatic and demographic characteristics.

The total human population, *N*, was assumed to be constant with the per capita death rate $\mu$N and the birth rate equals the death rate. This population is divided into five, susceptible (*S*), exposed (*E*), asymptomatic infected (*M*), symptomatic infected (*I*), and recovered (*R*). Starting the dynamics with the population of susceptible humans, *S*, they have never been infected by the dengue virus and are not immune to it. From the moment a susceptible human is bitten by a female mosquito infected with the dengue virus, $F_I$, this susceptible person may be exposed, *E*, given a transmission probability, $\psi$, and given the daily rate of humans bitten by infected females, $\xi$, relative to the total human population, *N*.

During the exposed or latent phase, *E*, the individual is infected but not yet infectious. So, this delay until the infection establishment causes the incorporation of this population into the classic SIR model. $\nu$ represents the intrinsic incubation rate of humans. If the exposed individual begins to show the symptoms caused by dengue, it passes to the symptomatic infected human phase *I*, given the fraction of symptomatic $\eta$. Otherwise, it moves to the stage of asymptomatic infected humans *M*, given the fraction $(1 - \eta )$. As there is no consensus in the literature on whether asymptomatic infected individuals are more infectious than symptomatic infected individuals and vice versa^9,12^, for simplicity, they are assumed to be equally infectious. Finally, the last human population is the recovered population *R*, composed of individuals who were infected after the viremia stage, given the recovery rates $\theta _I$ and $\theta _M$ of the symptomatic and asymptomatic infected, respectively.

Regarding the population dynamics of the vector *Ae. aegypti*, we consider four different stages. Firstly, *A* represents the immature phase composed of eggs, larvae, and pupae. The following three populations represent the female mosquito in the winged phase *F*, already in the oviposition phase: susceptible ($F_S$), exposed ($F_E$), and infected adult females ($F_I$). The number of larvae increases with the hatching of viable eggs, which the adult females deposit in breeding grounds available in nature. The parameter $\epsilon$ represents the production capacity of larvae from the fraction of viable eggs, which the female population will deposit in potential breeding sites, given the oviposition rate $\phi$. The nonlinear term $\left( 1 - \dfrac{A}{C}\right) F$ is a logistic type factor, which promotes a reduction in the oviposition rate per female unit according to the conditions of the breeding sites for receiving the larvae that hatch from the viable eggs. *C*, the carrying capacity, is the total breeding capacity associated with abundant nutrients and space, among others. Once the populations of eggs, larvae, and pupae were brought together in the immature population *A*, they were constrained in carrying capacity because of limited resources and intraspecific competition. If we did not establish this saturation, the immature population could grow indefinitely, which would be absurd given the biological limits mentioned for this growth.

The number of pupae decreases given the development rate represented by $\alpha$, when they finally pass into the adult phase of susceptible females, $F_S$, which are the ones that can contract the virus after biting an infected human. Among adult mosquitoes, the fraction corresponding to females is $\sigma$, resulting from the assumption that there are enough male mosquitoes for mating. The adult stage of females exposed to the dengue virus, $F_E$, grows by the encounter between susceptible females, $F_S$, and infected humans, *M* or *I*, given a probability of dengue virus transmission from human to mosquito, represented by $\beta$, and by the rate $\xi$ of humans bitten by mosquitoes daily, concerning the total human population, *N*.

In case of infection, exposed females still cannot transmit the virus, as they are in the extrinsic incubation period given by $\frac{1}{\gamma }$. After this period, the females culminate in the last stage, represented in the model as $F_I$, now corresponding to infected adult females. The per-capita natural mortality rates of the immature and adult populations are given by $\mu _A$ and $\mu _F$. The additional mortality, by chemical control of larvicides and adulticides, is given by $u_A$ and $u_F$, respectively.

Finally, the proposed model is described in Eq. (1):

1 $$\left\{ {\begin{array}{*{20}l} {\frac{{dS}}{{dt}} = \mu N - \frac{{\xi \psi SF_{I} }}{N} - \mu S} \hfill \\ {\frac{{dE}}{{dt}} = \frac{{\xi \psi SF_{I} }}{N} - (\nu + \mu )E} \hfill \\ {\frac{{dM}}{{dt}} = (1 - \eta )\nu E - (\theta _{M} + \mu )M} \hfill \\ {\frac{{dI}}{{dt}} = \eta \nu E - (\theta _{I} + \mu )I} \hfill \\ {\frac{{dR}}{{dt}} = \theta _{M} M + \theta _{I} I - \mu R} \hfill \\ {\frac{{dA}}{{dt}} = \varepsilon \phi \left( {1 - \frac{A}{C}} \right)F - (\alpha + \mu _{A} + u_{A} )A} \hfill \\ {\frac{{dF_{S} }}{{dt}} = \sigma \alpha A - (\mu _{F} + u_{F} )F_{S} - \frac{{\beta \xi F_{S} (M + I)}}{N}} \hfill \\ {\frac{{dF_{E} }}{{dt}} = \frac{{\beta \xi F_{S} (M + I)}}{N} - (\gamma + \mu _{F} + u_{F} )F_{E} } \hfill \\ {\frac{{dF_{I} }}{{dt}} = \gamma F_{E} - (\mu _{F} + u_{F} )F_{I} ,} \hfill \\ \end{array} } \right.{\text{ }}$$

in which $\mu , N, \xi , \psi , \eta , \nu , \epsilon , \theta _M, \theta _I, \phi , C, \gamma , \sigma , \alpha , \beta , \mu _{A}, \mu _{F}, u_A, u_F \in \mathbb {R}^{+}$ and $F_S + F_E + F_I = F$.

The regions of the system Eq. (1) with biological sense are defined by:

2 $$\begin{aligned} \Gamma _1 = \{(A, F_S,F_E,F_I) \in \mathbb {R}^{4}\mid A, F \geqslant 0 ~ \text {and} ~ 0 \leqslant A \leqslant C\} \quad ~ \text {and} \quad ~ \Gamma _2 = \{(S,E,M,I,R) \in \mathbb {R}^{5}\mid 0< S,E,M,I,R < N\}, \end{aligned}$$

that is, the populations are nonnegative, the immature stage population *A* does not exceed the carrying capacity *C* of the medium, and the human populations do not exceed the total number *N* of humans. By the next generation matrix method^13^ (see Supplementary Information), the expressions found for the basic offspring number, $Q_{0}$, and the basic reproduction number, $R_0$, are:

3 $$\begin{aligned} Q_{0}= & {} \frac{\sigma \alpha }{(\alpha + \mu _{A} + u_{A})}\frac{\varepsilon \phi }{(\mu _{F} + u_{F})} \quad \text {and} \quad R_0 = \frac{\gamma \xi \psi }{(\gamma + \mu _{F} + u_{F})(\mu _{F} + u_{F})}\nonumber \\{} & {} \times \left[ \frac{\beta \xi \bar{F_{S}} \eta \nu }{(\nu + \mu )(\theta _I + \mu )N} + \frac{\beta \xi \bar{F_{S}}(\eta - 1)\nu }{(\nu + \mu )(\theta _M + \mu )N}\right] . \end{aligned}$$

Considering the values of the epidemiological parameters of this system invariant in time and assuming the existence of the mosquito population, two equilibrium points can be determined, given by:

1. $P_0=(N,0,0,0,0,\bar{A},\bar{F_S},0,0)$, which is the trivial or disease-free equilibrium point, $\bar{A}=C\left( 1-\frac{1}{Q_{0}}\right)$ and $\bar{F_S}= \frac{\sigma \alpha }{\mu _{F} + u_F}\bar{A}$. In this case, both the mosquito and human populations are free of the dengue virus.
2. $$P_1=(S^{**},E^{**},M^{**},I^{**},R^{**}, A^{**},F_S^{**}, F_E^{**}, F_I^{**})$$, which is the nontrivial equilibrium or epidemic equilibrium point, i.e., when the dengue virus is present. In this case,
  $$\begin{aligned} S^{**}= & {} \dfrac{\mu N}{\xi \psi \dfrac{F_I^{**}}{N}+\mu }, E^{**} = \dfrac{\xi \psi F_I^{**} \mu N (\nu + \mu )}{\xi \psi F_I^{**}+\mu N}, M^{**} = \dfrac{(1 - \eta ) \nu \xi \psi F_I^{**} \mu N (\nu + \mu )}{(\theta _M + \mu ) (\xi \psi F_I^{**}+\mu N)},\\ I^{**}= & {} \dfrac{\eta \nu \xi \psi F_I^{**} \mu N (\nu + \mu )}{(\theta _I + \mu ) (\xi \psi F_I^{**}+\mu N)}, R^{**} = \dfrac{\theta _M M^{**} + \theta _I I^{**}}{\mu }, A^{**} = C - \dfrac{C(\alpha + \mu _{A} + u_A)(\mu _{F} + u_F)}{\sigma \alpha \epsilon \phi },\\ F_S^{**}= & {} \dfrac{\sigma \alpha C - \left[ \dfrac{C(\alpha + \mu _{A} + u_A)(\mu _{F} + u_F)}{\epsilon \phi } \right] }{\left[ (\mu _{F} + u_F) + \dfrac{\beta \xi (M+I)}{N}\right] }, F_E^{**} = \dfrac{\beta \xi F_S^{**}(M+I)}{N(\gamma +\mu _{F} + u_F)}, \quad \text {and} \quad F_I^{**} = \dfrac{\gamma F_E^{**}}{N(\mu _{F} + u_F)}. \end{aligned}$$

The disease-free equilibrium point, $$P_0=(N,0,0,0,0,\bar{A},\bar{F_S},0,0)$$, is locally asymptotically stable for $$0< R_0 < 1$$ and unstable for $$R_0 > 1$$. On the other hand, the epidemic equilibrium point, $$P_1=(S^{**},E^{**},M^{**},I^{**},R^{**}, A^{**} ,F_S^{**}, F_E^{**}, F_I^{**})$$, is locally asymptotically stable for $$R_0 > 1$$ and unstable for $$0< R_0 < 1$$. Stability analysis demonstrations are also in the Supplementary Information. Furthermore, we use the fourth-order Runge–Kutta method for computational simulation of the dynamical system Eq. (1), with step $$\Delta = 0.01$$.

### Adjusting the climate-dependent parameters

Table 1 contains the interpretation of all model parameters, showing those that vary over time, their acceptable ranges according to the literature, and those constant or climate-independent. Next, we explain how the parameterization was performed according to each climate variable. To visualize the variation of these parameters in time, see Supplementary Fig. S2 online.

**Table 1:** The model parameters.

Symbol	Description	Range	Value	Units	Source
*N*	Total human population	–	$2.5\times 10^{6}$	People	^14^
$\mu$	Natural death rate of human	$3.9\times 10^{-5}$–$4.6\times 10^{-5}$	$3.9\times 10^{-5}$	$\text {Days}^{-1}$	^15,16^
$\xi$	Rate of humans bitten daily	0.3–1	0.8	$\text {Days}^{-1}$	^17,18^
$\psi$	Probability of dengue virus transmission from mosquito to human	0.1–0.75	0.75	Dimensionless	^19,20^
$\beta$	Probability of dengue virus transmission from human to mosquito	0.5–1	0.75	Dimensionless	^15,21^
$\nu$	Human intrinsic incubation rate	0.1–0.33	0.1	$\text {Days}^{-1}$	^19,22^
$\eta$	Fraction of symptomatic infected humans	0.2–0.6	0.25	Dimensionless	^9,23^
$\theta _M$	Recovery rate of dengue virus for asymptomatic infected humans	0.05–0.4	Via model fit	$\text {Days}^{-1}$	^18,24^
$\theta _I$	Recovery rate of dengue virus for symptomatic infected humans	0.083–0.25	Via model fit	$\text {Days}^{-1}$	^15,25^
$\epsilon$	Fraction of eggs hatching	0–1	0.5	Dimensionless	^16,26^
$\sigma$	Among adult mosquitoes, fraction corresponding to females	0–1	0.5	Dimensionless	^16,26^
*k*	Ivanov model constant	$3.3\times 10^{-6}$–$6.6\times 10^{-5}$	$5.8\times 10^{-5}$	mm/$^{\circ }$ C$^2$	^27^
*m*	Scale factor	–	1	Dimensionless	Assumed
$H_{max}$	Maximum amount of water available in the breeding site	15–30	24	mm	^27^
*C*(*P*, *T*, *H*)	Carrying capacity	1–$7.5\times 10^{6}$	–	Dimensionless	^28^
$\phi (T)$	Oviposition rate by each female	0.3548–9.57	–	$\text {Days}^{-1}$	^29^
$\alpha (T)$	Development rate of immatures to adults	0.011–0.158	–	$\text {Days}^{-1}$	^29^
$\gamma (T)$	Extrinsic incubation rate of mosquitoes	0–0.1087	–	$\text {Days}^{-1}$	^22,30,31^
$\mu _{A}(P,T)$	Natural death rate of eggs, larvae and pupae	0.01–0.2616	–	$\text {Days}^{-1}$	^29,32^
$\mu _{F}(T)$	Natural death rate of female mosquitoes	0.026–0.043	–	$\text {Days}^{-1}$	^29^
$u_{A}$	Investment of control in the immature stage	–	Via optimization	Kilograms	–
$u_{F}$	Investment of control in the adult stage	–	Via optimization	Liters	–

#### Precipitation-dependent entomological parameter

It is known that the influence of rain is difficult to assess, especially during outbreaks of vector-borne diseases^33,34^. On the other hand, many authors agree that precipitation directly influences the aquatic stages of the vector *Ae. aegypti*, especially in the development of the larvae. They can reach the adult stage because minimal contact with water is required for this development process. However, there is also the phenomenon that the breeding sites can be washed away if there is a high incidence of rain, which eliminates the aquatic phases of the breeding sites and interrupts the reproductive cycle^33,35^. Thus, only the influence of precipitation on mortality in the aquatic phase, $\mu _A$, was considered. The parameterization was done linearly, according to the expression below, adapted from^36^.

4 $$\begin{aligned} \mu _A(P) ={\mu _A}_{min} + ({\mu _A}_{max} - {\mu _A}_{min} )\left( \dfrac{P - P_{1}}{P_{2} - P_{1}}\right) , \end{aligned}$$

in which $P = P(t)$ is the daily precipitation; $P_{1}(t)$ and $P_{2}(t)$ represent, respectively, the lowest and highest precipitation values during the study period; ${\mu _A}_{min}$ and ${\mu _A}_{max}$ represent, respectively, the minimum and maximum value of $\mu _A$, according to data in the literature. In addition to precipitation, $\mu _A$ is also influenced by temperature, as described below.

#### Temperature-dependent entomological parameters

In the literature, it is possible to find that temperature influences some of the entomological parameters^29,33,37^. For example, the authors^29^ conducted temperature-controlled laboratory experiments to see under what temperature conditions vector populations thrive. Thus, they established a polynomial for each entomological parameter influenced by temperature, minimizing the adjustment error by the linear least squares method.

In this work, the influence of temperature is expressed through the function $\theta (T)$, Eq. (5). It represents the polynomial fit made for each temperature-dependent parameter $\theta _j(T)$, according to the experiments done by^29^. For the adjustment according to the temperature of each parameter, a polynomial of degree $\kappa$ is given by:

5 $$\begin{aligned} \theta _j(T) = \sum _{i=0}^{\kappa _j}b_{i_j}T^{i}, \end{aligned}$$

in which $\theta _1(T), \ldots , \theta _4(T)$ are the temperature-dependent components of $\phi$, $\alpha$, $\mu _A$, and $\mu _F$, respectively; *T* is the average daily temperature (in $^{\circ }$C), and the coefficients $b_{i_j}$, with $i=0, 1, 2, \dots , \kappa _j$ and $j=1, 2 , \dots , 4$, are fitted by the usual linear least squares method. Table 2 shows the values found for each coefficient $b_{i_j}$ of the polynomial, with units $\hbox {days}^{- 1}$($^{\circ }$C)$^{- i }$, The degrees $\kappa _j$ are set to 4 for parameters $\phi , \mu _A$, and $\mu _F$ and set to 7 for parameter $\alpha$.

**Table 2:** Model temperature-dependent coefficients parameters^29^.

Symbol	$b_{0}$	$b_{1}$	$b_{2}$	$b_{3}$	$b_{4}$
$\phi$	$-5.400$	1.800	$-2.124\times 10^{-1}$	$1.015\times 10^{-2}$	$-1.515\times 10^{-4}$
$\mu _{A}$	2.130	$-3.797\times 10^{-1}$	$2.457\times 10^{-2}$	$-6.778\times 10^{-4}$	$6.794\times 10^{-6}$
$\mu _{F}$	$8.692\times 10^{-1}$	$-1.590\times 10^{-1}$	$1.116\times 10^{-2}$	$-3.408\times 10^{-4}$	$3.809\times 10^{-6}$
$\alpha$	$1.310\times 10^{-1}$	- $5.723\times 10^{-2}$	$1.164\times 10^{-2}$	$- 1.341\times 10^{-3}$	$8.723\times 10^{-5}$
	$b_{5}$	$b_{6}$	$b_{7}$	–	–
	$-3.017\times 10^{-6}$	$5.153\times 10^{-8}$	$-3.420\times 10^{-10}$	–	–

In the specific case of mortality in the aquatic phase, $\mu _A$, which is influenced by temperature and precipitation^3,10^, the dependency can assume a well-behaved function, so that $\mu _A(P,T)=a_0 + a_1 \mu _A(P) + b_1 \mu _A(T) + O(P^2,T^2)$^11^. For simplicity, as a working hypothesis, and because the model (daily) rates are typically less than one, we retain the linear part for the association. It complies with the literature values, respecting the range shown in Table 1. The value is then defined here by the average values obtained in Eqs. (4) and (5).

Temperature can also help spread arboviruses. At higher temperatures, infected females become infectious through viral shedding at a significantly faster rate^37^, i.e., the higher the temperature, the higher the rate $\gamma$ and the shorter the time required to transmit the virus. Thus, Focks et al.^38^ showed this relationship with temperature between 12 and 36 $^{\circ }$C. Hence, in this work, the following parameterization was used as a function of temperature for $\gamma$^31^:

6 $$\begin{aligned} \gamma (t) = -0.1393 + 0.008T(t). \end{aligned}$$

#### Precipitation, temperature, and humidity-dependent entomological parameter

Many interpretations of carrying capacity, *C*, refer exclusively to the availability of resources in terms of substances necessary for the species to live and reproduce. However, this amount can be understood from a broader point of view as the environmental potential of the species. An example is the enormous capacity of a breeding site full of water and nutrients to produce mosquitoes from larvae at 25 $^{\circ }$C. Adult females lay above the water level inside the breeding grounds or where they may be flooded^39,40^. With sufficient rain and favorable humidity conditions, the eggs hatch, generating new larvae^41^.

Thus, precipitation supplies breeding sites and originates new breeding sites in which the larvae develop before becoming adult mosquitoes. However, evaporation tends to reduce these breeding sites. Therefore, due to the strong influence that the amount of water available in breeding grounds and puddles from rain exert on the carrying capacity, also associated with temperature and humidity, it was considered in this work that *C* varies over time.

How the parameterization of the carrying capacity took place was based on^27^, as defined below:

7 $$\begin{aligned} C(t) = C_{max} \frac{H(t)}{H_{max}} + 1, \end{aligned}$$

in which $C_{max}$ is the maximum value that all breeding sites can assume. Following the reasoning of^28^, usually, this value corresponds to three times the size of the human population.

Like the carrying capacity depends on the amount of water available, *H*(*t*), its variation is given as follows:

8 $$\begin{aligned} H(t+1) = {\left\{ \begin{array}{ll} 0 &{} \text {if} \, H(t) + \Delta (t) \leqslant 0\\ H_{max} &{} \text {if} \, H(t) + \Delta (t) \geqslant H_{max}\\ H(t) + \Delta (t) &{} \text {otherwise,} \\ \end{array}\right. } \end{aligned}$$

in which $H_{max}$ is the maximum amount of water a breeding site can store without overflowing; $\Delta (t) = P(t) - Evap(t)$; *P*(*t*) represents the daily precipitation, and *Evap*(*t*) is the daily evaporation rate. This rate, in turn, is given by the following expression, adapted from the Ivanov^42^ model:

9 $$\begin{aligned} Evap(t) = \cos (k(25\,^{\circ }\text {C} + T(t))^2 (100 - Hum(t)))m, \end{aligned}$$

in which *T* is the average daily temperature, *Hum* is the daily humidity, *k* represents the Ivanov model constant, and *m* is a scale factor. This model also considers evapotranspiration, which differs from evaporation because it evaluates the transfer of water to the atmosphere from the soil and plants’ transpiration. Although the practical part of measurements related to evapotranspiration is complex^43^, the Ivanov model is commonly used in the literature due to its lower complexity than other methods^27,44,45^. The cosine function represents the epidemic cycle and seasonal influence on dengue cases during the study period.

### Multiobjective optimization model

Multiobjective optimization deals with problems that contain at least two objective functions. Usually, these functions conflict with each other in such a way that improving one of them can lead to the degradation of others. As the model Eq. (1) is generic, in the sense that it could be applied to any disease transmitted by the mosquito *Ae. aegypti* in any city, a trade-off between the cost of vector control could be portrayed by the function $J_{1}(u_A,t_A,u_{F},t_{F})$ versus the hospital cost demanded to treat infected humans, represented in the function $J_{2}(u_A,t_A,u_{F},t_{F})$. Thus, Eq. (10) represents a general formulation of the multiobjective optimization problem, which is subject to the dynamic system Eq. (1). The problem could also impose other constraints, for example, the minimum and maximum amount of control investment and minimum and maximum time of control application.

10 $$\begin{aligned} \begin{aligned} \min _{u_A,u_F} \left\{ \begin{array}{lll} J_{1}(u_A,t_A,u_{F},t_{F}) = s_{1} \int _{t_{A}}^{t_{A} + \tau _A} {u_{A}}dt \,+\, s_{2} \int _{t_{F}}^{t_{F} + \tau _F} {u_{F}}dt \\ \\ J_{2}(u_A,t_A,u_{F},t_{F}) = s_{3}\int _{0}^{t_{I}}I dt, \end{array}\right. \end{aligned} \end{aligned}$$

where $s_{1}$ is the relative cost with control in the immature phase of the vector; $u_{A}$ is the intensity of additional larvicides to be applied in the immature phase, starting at time $t_{A}$ until any time $t_{A} + \tau _A$; $s_{2}$ the relative cost with control in the adult phase; $u_F$ is the intensity of insecticides to be applied in the adult phase, starting at time $t_{F}$ until any time $t_{F} + \tau _F$; $s_{3}$ is the hospital cost of infected humans in any time $t_{I}$; and *I* corresponds to the symptomatic infected population, which demands treatment costs.

The search for candidate solutions is carried out in the decision variable space in optimization problems. Due to the characteristic of a multiobjective problem being composed of more than one objective function, this process is quite different. When evaluating these functions in the search for candidate solutions, the decision variables will be mapped from the decision variable space to a new search space associated with the objective functions, which are vectors located in the called objective space. This mapping to the objective space is complex and depends on the functions and constraints of the multiobjective problem. Two compromise solutions that are close in the decision variable space will not necessarily be close in the objective space.

Let *X* be the decision variable space and let $\mathscr {F}_X$ be the objective space. Let the vector *J* be described component-wise by $J = (J_1,J_2)$ and let the set $\mathscr {F}_Y$ represent the image set of region $\mathscr {F}_X$. The point $\bar{x}_1 \in \mathscr {F}_X$ is said to be dominated if there is another point $\bar{x}_2 \in \mathscr {F}_X$ that is not worse than $\bar{x}_1$ for all objectives, as the following relation shows^2^:

11 $$\begin{aligned} J_1(\bar{x}_1)\le J_2(\bar{x}_2) \, \text {and} \, J_1(\bar{x}_1) \ne J_2(\bar{x}_2). \end{aligned}$$

A feasible solution is said to be nondominated if it is not dominated by any other feasible solution in *X*. A Pareto-optimal solution is a vector $x \in \mathscr {F}_x$ concerning the objective space. The set of all Pareto-optimal solutions is called Pareto-optimal set. The image of the Pareto-optimal set in the objective space is called Pareto front^2^.

In this work, we use the Non-dominated Sorting Genetic Algorithm II (NSGA-II)^46^ as an optimization algorithm to find solutions for the problem. This optimization algorithm classifies and organizes the compromise solutions in nondominated fronts. The solutions in the first front correspond to the best solutions, while the last contains the worst ones. There is no dominance between the solutions located on the same front, but the following fronts contain results that favor less the natural trade-off relationship of the problem. So, it will be necessary to run the optimization algorithm several times to estimate the Pareto-optimal set. The result will be a set of combined nondominated feasible solutions. Table 3 describes the parameters used for the simulations performed with the NSGA-II. The stopping criterion was when the algorithm reached the maximum number of generations.

**Table 3:** Algorithmic parameters used in running the NSGA-II.

Parameter	Value
Number of runs	30
Maximum number of generations	2000
Population size	500
Recombination rate	90%
Mutation rate	5%

In summary, there are two main goals in multiobjective optimization: first, to find the set of nondominated solutions in equilibrium with all involved objectives, and second, to find the most diverse solutions possible, ensuring that they are sparsely spaced in the Pareto-optimal region^47^. The solution that will actually be implemented depends on the choice of one or more decision-makers. In this case, public health managers are responsible for decision-making regarding allocating resources for vector control and reducing the risk of exposure of a given population to a virus and the costs arising from probable hospitalization.

In conclusion, the multiobjective approach is equivalent to varying scenarios. Each point on the Pareto front is a trade-off between control interventions and hospital cost, achieved without the need for massive optimization tests to vary control amounts or control application start time. It is up to the decision-maker to choose the point that will best meet its needs concerning the costs involved. In general, for each level of financial resources available, the method finds additional control associated with the respective reduction in the number of infected people and hospital costs.

### Study area

**Figure 1:**
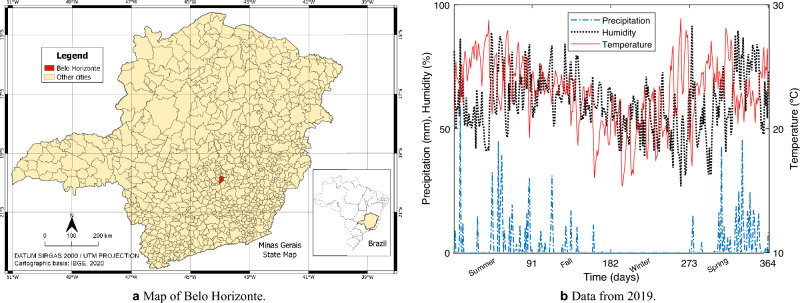
Location of the city of Belo Horizonte in the State of Minas Gerais and its series of precipitation, temperature, and humidity. The map was created using QGIS (QGIS Development Team, software version 3.16.13, https://www.qgis.org).

As a case study, we use the model Eq. (1) in the computational simulations and formulate a multiobjective optimization problem to control the vector *Ae. aegypti* in the city of Belo Horizonte, the capital of the State of Minas Gerais, Brazil. It is located at latitude 19$^{\circ }$ 48$^{\prime }$ 57$^{{\prime }{\prime }}$ S and longitude 43$^{\circ }$ 57$^{\prime }$ 15$^{{\prime }{\prime }}$ W, as shown in Fig. 1a^48^. The city has an area of approximately 331 km$^{2}$ and is located 716 km away from Brasília, the Federal Capital.

According to the last census data, the population in 2010 was 2,375,151 inhabitants, and the population in 2021 was estimated at 2,530,701 inhabitants^14^. The biome of the city is the Atlantic forest and the humid temperate climate with a dry season (Köppen: *Cwb*^49^), that is, with a dry winter, hot, and humid summer. Figure 1b shows the city’s series of precipitation, temperature, and humidity during 2019 using the data provided by the National Institute of Meteorology (INMET^50^). The annual average temperature is around 21 $^{\circ }$C, favoring *Ae. aegypti* reproduction.

**Figure 2:**
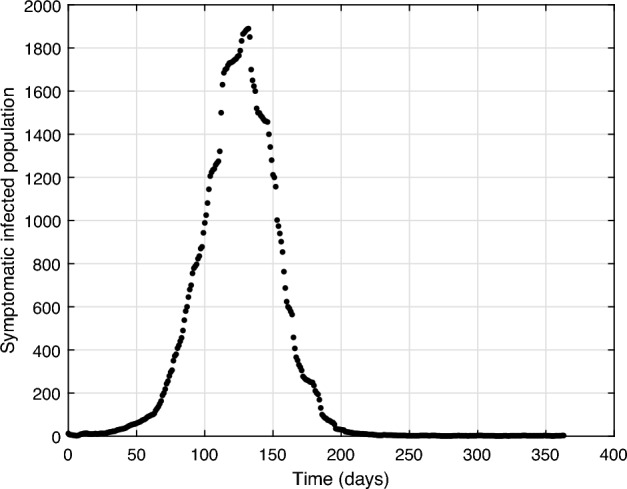
Number of reported symptomatic dengue virus infections in Belo Horizonte in 2019.

Official data on those infected with classic dengue, dengue with signs of alarm, and severe dengue are provided by^51^ in epidemiological weeks. Analyzing the historical series of Belo Horizonte since 2010, every 3 years, there has been a dengue epidemic, with the years with the highest number of infected people being 2010 (51,813 cases), 2013 (97,982 cases), 2016 (156,542 cases) and 2019 (116,320 cases). This case study is interested in data on actual dengue infections in Belo Horizonte, especially in epidemic years, to adjust the model with data from the last epidemic that occurred in 2019. However, the vector control actions are daily, and it was decided to follow this time scale for standardization. The distribution of daily cases of the last dengue epidemic in the city is shown in Fig. 2.

### Control measures in Belo Horizonte

Following the Brazilian Ministry of Health assumptions, Belo Horizonte City Hall carried out two types of control applications yearly. First, the focal treatment, which we will call $u_A$, consists of applying larvicide whenever necessary by endemic diseases combat agents. Homes are visited every 2.5 months, as the drug has a residual effect of 2 months, so five cycles of larvicide control application ($u_{A}$) are performed per year. Second, the perifocal treatment, which we will call $u_{F_{1}}$, is an adulticide applied whenever necessary at strategic points, with biweekly visitation and a residual effect of 2 months as well. Moreover, a third way to reduce the number of mosquitoes, although it is not considered a form of vector control, but blocking transmission, is the treatment of ultra-low volume (ULV) spraying that we will call $u_{F_{2}}$ (because it also causes a reduction in female population). ULV is applied only when there are many suspected or confirmed dengue cases. The product has virtually no residual effect, as when it is applied, only mosquitoes in flight will be affected.

As we will show later, such control actions were insufficient to contain the 2019 epidemic. Therefore, it was decided to simulate in this work an additional control approach to investigate, in the city of Belo Horizonte, possible improvements in the performance of the fight against *Ae. aegypti* in epidemic years. In practice, the additional control approach consists of applying an additional quantity of kilograms of larvicides ($u_A$) and liters of adulticides ($u_F = u_{F_{1}} + u_{F_{2}}$ ). However, some particularities need to be explained about the additional control approach. First, the larvicide application will be explained, then control via the perifocal application and, shortly after, the ULV spraying.

The proposal is to maintain the periodicity for larvicide control application ($u_A$) because the Brazilian Ministry of Health recommends it. So, the duration of each cycle was limited to 73 days, except for the last cycle, which was left with 72 days, totalizing 364 days. In this way, the control cycles follow the same 2.5-month interval established by the Belo Horizonte City Hall. The differential of the proposal is to allow the optimization algorithm to search for the best moment to start applying additional control in each cycle and to define the amount of kilograms demanded.

For perifocal control ($u_{F_{1}}$), there is no information on how many cycles are performed per year on average. So, for simplicity, the same number of cycles as the larvicide control was defined for the $u_{F_{1}}$ control. That is, both are performed at the same instant of time ($t_{A} = t_{F_1}$), representing the work of the same endemic diseases combat agent.

It remains to define the proposal for ULV spraying ($u_{F_{2}}$). There needs to be more information on how many control cycles are carried out per year, as it is carried out on demand based on an outbreak in the number of dengue cases. So, in this work, the estimated number of mosquitoes will be considered. As expected, the most significant number of mosquitoes is found when infected humans peak. From the model fitting, the application time that coincides with the peak formed between days 50 and 200 was defined for the search space of the optimization algorithm.

### The need for additional control

By Eq. (3), $R_0$ explicitly depends on $u_{F}$, as it also depends on $\bar{F_S}$, which in turn is influenced by both additional control parameters, $u_{A}$ and $u_{F}$. Also consider that $u_F = u_{F_{1}} + u_{F_{2}}$. Therefore, it can be inferred that $R_0$ is influenced by vector control actions. Thus, we sought to establish a relationship between $R_0$ and the model’s additional control parameters in such a way as to represent the control intervals that will guarantee $R_0(u_A,u_F) < 1$ and $R_0(u_A,u_F ) = 1$, that is, no epidemic.

**Figure 3:**
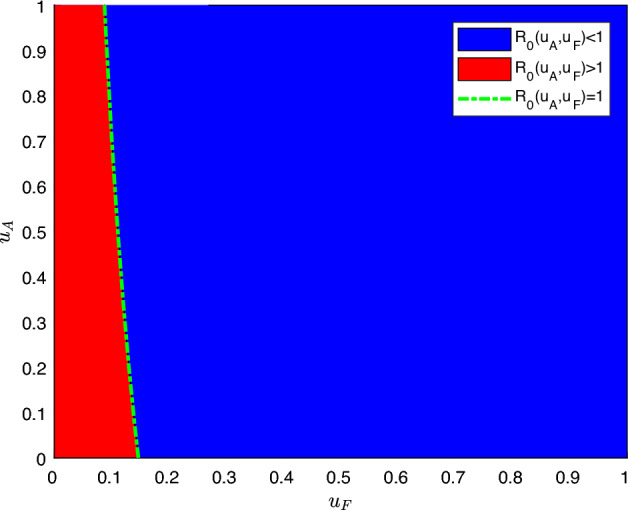
Relationship between the controls and the basic reproduction number. For $u_A$ and $u_F$, the range from 0 to 1 was considered as the percentage of additional control: the amount of larvicide applied in kilograms, and the amount of adulticide applied in liters, respectively. The blue region (on the right) indicates ordered pairs, $(u_A,u_F)$, where $R_0(u_A,u_F) < 1$, while the red region (on the left) indicates ordered pairs $(u_A,u_F)$, where $R_0(u_A,u_F) > 1$ and therefore there is an epidemic. The green dashed line indicates ordered pairs, $(u_A,u_F)$, where $R_0(u_A,u_F) = 1$.

Before presenting this relationship, it is worth noting that climatic variables influence some model parameters over time. However, this variation is not considered to establish the relationship between the controls and the basic reproduction number. Hence, for this step, it was necessary to fix all parameters. So, we computed the climate-dependent parameter’s values at each instant of time during the 364 days of the study horizon. Then, we extracted the average of each one of them to bring a more reliable perspective of $R_0$ because insecticide control is used even when there is no dengue outbreak. Finally, those fixed values were used to establish the relationship shown in Fig. 3.

According to Fig. 3, as there is a red region in which $R_0(u_A,u_F) > 1$, it is possible to infer that those actions to combat the vector *Ae. aegypti* already held in the city were not enough to prevent a dengue epidemic. Therefore, it is necessary to study the combination of controls that will at least guarantee $R_0(u_A,u_F) = 1$, in which there is no epidemic. In this sense, Fig. 3 shows that larvicidal control alone is not enough to prevent an epidemic. On the other hand, the application of more adulticide control would be enough to maintain $R_0(u_A,u_F) = 1$. This information is essential for defining the amount of the needed additional control actions described below.

### Control modeling in Belo Horizonte

We consider two distinct ways to model the control in the numerical simulations, one for the aquatic control $u_{A}$ and the perifocal control $u_{F_{1}}$ and another for the ULV spraying $u_{F_{2} }$. Firstly, the descending control was considered for the aquatic phase control and the perifocal control because they have a residual effect of approximately 2 months. This type of descending control is based on the control amount $u_{A}$ in kilograms and the control amount $u_{F_{1}}$ in liters. These amounts are reduced at each instant of time, simulating the residual effect of the focal and perifocal treatments’ insecticides. So, the control starts at a specific instant of time ($t_0$) defined by the optimization algorithm, and its effect can still be observed in the following days ($t_0 + \tau$, with $\tau = (\tau _A, \tau _{F_{1}}) = 60$). Therefore, the control action in the last cycle must start no later than day 304 so that all cycles begin and end in the same year.

**Figure 4:**
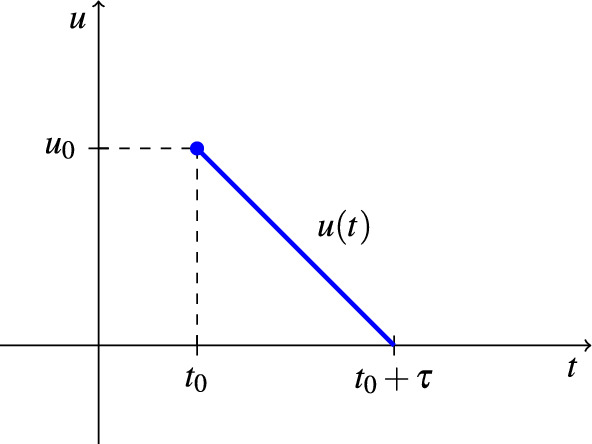
Example of descending control. The control application $u(t)=(u_A(t),u_{F_{1}}(t))$ is performed from time $t_0 = (t_{A},t_{F_{1}})$ until time $t_0 + \tau$, with $\tau = (\tau _A, \tau _{F_{1}})$.

An illustration to exemplify the type of descending control is shown in Fig. 4. In it, $u(t)=(u_A(t),u_{F_{1}}(t))$ because, as stated before, the two controls are carried out simultaneously by the same endemic diseases combat agent.

Thus, by the equation of a line, the descending control is formally defined as:

12 $$\begin{aligned} u(t) = -\frac{u_{0}}{\tau } t + \frac{u_{0}}{\tau } (t_0 + \tau ). \end{aligned}$$

**Figure 5:**
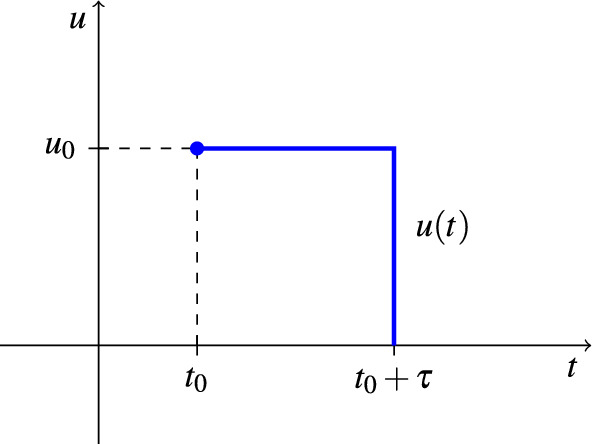
Example of step size control. The control application $u(t)=u_{F_{2}}(t)$ is performed from time $t_0= t_{F_{2}}$ until time $t_0 + \tau$, with $\tau = \tau _{F_{2}}$.

In the case of the ULV, the application lasts only 1 day since there is no residual effect on the ULV spraying. Therefore, we considered the step size control represented in Fig. 5. It allows the application of the chemical $u_{F_{2}}$ in liters, starting at the instant of time $t_0$ and ending at the time $t_0 + \tau$ (with $\tau = \tau _{F_{2}} = 1$), that is, the duration of the treatment will be limited to 1 day. The optimization algorithm is responsible for choosing the best day for the application.

Finally, we can present the multiobjective formulation of the problem of this case study. We consider the trade-off in minimizing the number of females *Ae. aegypti* with the lowest intensity of control possible so that there are fewer people susceptible to contracting the dengue virus and, at the same time, a lower public hospital cost for those infected. Thus, Eq. (13) represents a general formulation of the multiobjective optimization problem, subject to the constraints of the dynamical system and the bounds of the decision variables in Eq. (14).

13 $$\begin{aligned}{} & {} \begin{aligned} \min _{u_A^{i},u_F^{i}} \left\{ \begin{array}{lll} J_{1}(u_A^{i},t_A^{i},u_{F_1}^{i},t_{F_1}^{i},u_{F_2}^{i},t_{F_2}^{i}) = s_{1} \int _{t_{A}^{i}}^{t_{A}^{i} + \tau _A} {u_{A}^{i}}dt \,+\, s_{2} \int _{t_{F_{1}}^{i}}^{t_{F_{1}}^{i} + \tau _{F_{1}}} {u_{F_1}^{i}}dt \, +\, s_{3} \int _{t_{F_{2}}^{i}}^{t_{F_{2}}^{i} + \tau _{F_{2}}} {u_{F_2}^{i}}dt\\ \\ J_{2}(u_A^{i},t_A^{i},u_{F_1}^{i},t_{F_1}^{i},u_{F_2}^{i},t_{F_2}^{i}) = s_{4}\int _{0}^{364}I dt, \end{array}\right. \end{aligned} \end{aligned}$$

14 $$\begin{aligned}{} & {} \begin{aligned} \text {subject to:} \left\{ \begin{array}{lll} \dfrac{dS}{dt}=\mu N - \dfrac{\xi \psi S(t)F_I(t)}{N} -\mu S(t)\\ \dfrac{dE}{dt}=\dfrac{\xi \psi S(t)F_I(t)}{N} -(\nu + \mu ) E(t)\\ \dfrac{dM}{dt}=(1-\eta )\nu E(t) -(\theta _M + \mu ) M(t)\\ \dfrac{dI}{dt}=\eta \nu E(t) -(\theta _I + \mu ) I(t)\\ \dfrac{dR}{dt}=\theta _M M(t)+\theta _I I(t) - \mu R(t) \\ \dfrac{dA}{dt}= \epsilon \phi (T)\left( 1-\dfrac{A(t)}{C(P,T,H)}\right) F - (\alpha (T) + \mu _{A}(P,T) + u_A(t))A(t)\\ \dfrac{dF_S}{dt}= \sigma \alpha (T) A(t) - (\mu _{F}(T) + u_F(t))F_S(t) - \dfrac{\beta \xi F_S(t) (M(t)+I(t))}{N}\\ \dfrac{dF_E}{dt}= \dfrac{\beta \xi F_S(t) (M(t)+I(t))}{N} - (\gamma (T)+\mu _{F}(T) + u_F(t))F_E(t) \\ \dfrac{dF_I}{dt}= \gamma (T) F_E(t) - (\mu _{F}(T) + u_F(t))F_I(t)\\ 0 \leqslant u_{A}^{i} \leqslant 0.04; ~i = 1, 2, \ldots ,5\\ 0 \leqslant u_{F_1}^{i} \leqslant 0.02; ~i = 1, 2, \ldots ,5\\ 0 \leqslant u_{F_2}^{i} \leqslant 0.05; ~i = 1, 2\\ 1 \leqslant t_{A}^{1}, t_{F_1}^{1} \leqslant 73\\ 74 \leqslant t_{A}^{2}, t_{F_1}^{2} \leqslant 146\\ 147 \leqslant t_{A}^{3}, t_{F_1}^{3} \leqslant 219\\ 220 \leqslant t_{A}^{4}, t_{F_1}^{4} \leqslant 292\\ 293 \leqslant t_{A}^{5}, t_{F_1}^{5} \leqslant 304\\ 50 \leqslant t_{F_2}^{i} \leqslant 200; ~i = 1, 2. \end{array}\right. \end{aligned} \end{aligned}$$

in which $s_{1}$ is the relative cost with control in the immature stage; $s_{2}$ the relative cost with perifocal control and $s_{3}$ with ULV spraying, both in the adult stage; $s_{4}$ the public hospital cost of hospitalizations triggered by classic dengue, dengue with warning signs and severe dengue; $u_{A}$ refers to the intensity of additional larvicides to be applied in the immature phase, starting at time $t_{A}$; $u_F = u_{F_1} + u_{F_2}$ refers to the intensity of additional insecticides to be applied in the adult phase, starting at time $t_{F_1}$ for perifocal control, and at time $t_{F_2}$ for ULV spraying; *i* refers to each control application in each cycle; and *I* corresponds to the symptomatic infected population, which demands treatment costs. In this work, as the same endemic diseases combat agent applies larvicidal and perifocal control, $t_{A} = t_{F_1}$.

The monetary values of costs $$s_{1}$$, $$s_{2}$$, $$s_{3}$$, and $$s_{4}$$ will be described below. All costs mentioned in this work were initially calculated in the local currency (Real - R$) and converted to US dollars (US$) using an average exchange rate (R$ 1 = US$ 0.25 on December 31, 2019^52^). Starting with the control inputs and ULV spraying, the definition of these values took into account the data provided by the Belo Horizonte City Hall and by DataSUS^51^. In 2019, 592 kilograms of the Sumilarv® larvicide for focal treatment were passed on to the City Hall, each kilo costing US$ 19.77. In the same year, 21.5 l of a compound based on Bendiocarb were received for perifocal treatment, each liter costing US$ 4.20. Furthermore, 100 l of Malathion adulticide for ultra-low volume spraying were also passed on, each liter costing US$ 10.17. Thus, $$s_{1}$$ = US$ 19.77, $$s_{2}$$ = US$ 4.20, and $$s_{3}$$ = US$ 10.17 were defined.

Before presenting the value of $$s_{4}$$, which involves the hospital cost, it is necessary to consider the following information. In 2019, 2814 people were hospitalized, 2360 due to classic dengue and 454 due to severe dengue, dengue with warning signs or inconclusive^53^. Among the total infected in the same year, which was 116,320 people, the percentage that required hospitalization in public hospitals was 2.42%. The average cost per hospitalization in public hospitals was US$ 108.78 for classic dengue and US$ 123.22 for dengue with warning signs and severe dengue. Finally, to obtain an average hospital cost per infected person, we calculated:

15 $$ s_{4}  = \underbrace {{\left( {\frac{{2360}}{{2814}} \times 108.78} \right)}}_{{\begin{array}{*{20}c}    {{\text{Average cost of hospitalization}}}  \\    {{\text{for classic dengue}}}  \\   \end{array} }}\quad  + \underbrace {{\left( {\frac{{454}}{{2814}} \times 123.22} \right)}}_{{\begin{array}{*{20}c}    {{\text{Average cost of hospitalization}}}  \\    {{\text{for severe dengue and with warning signs}}}  \\   \end{array} }} \times \underbrace {{0.0242}}_{{\begin{array}{*{20}c}    {{\text{Number of infected}}}  \\    {{\text{people who need}}}  \\    {{\text{hospitalization}}}  \\   \end{array} }} = {\text{US}}\$ {\mkern 1mu} {\text{2}}.{\text{69 }} $$

Thus, each infected person cost Belo Horizonte US$ 2.69 in 2019 for public hospital treatment. Across the country, citizens’ public hospitalizations are free of charge and funded by the government’s Unified Health System (Sistema Único de Saúde—SUS).

Regarding the amount of additional control, following Fig. 3, if the amount of larvicide already applied by the city were doubled, it would still not be enough without the joint application of adulticide (perifocal treatment or ULV spraying). On the other hand, if an additional $20\%$ of adulticide control were applied all the time, for example, even without larvicide, it would guarantee the absence of an epidemic. As it is impracticable to apply control every day, we will allow, following the threshold, up to $20\%$ of an additional control for the control in the immature phase and the one in the adult phase. So, the two controls and the ULV spraying will be applied for an epidemic year. As they are the amount of additional control, in kilograms and liters, $u_{A}$ and $u_{F}$ are intrinsically the sums of the additional control intensity to be used in each cycle of each approach, as the optimization results. Hence, $u_{A} = \sum _{1}^{n}u_{A}^{i} \, \text {kilos}$ and $u_{F} = \sum _{1}^{n}u_ {F}^{i}\, \text {liters}$ $(u_F = u_{F_1} + u_{F_2})$, with *n* representing the number of cycles in each approach and $~i = 1, \ldots , n$ representing each cycle.

The values the control decision variables can assume in each cycle will now be discussed. In 2019, Belo Horizonte City Hall reported having spent 592 kilos of larvicide, and we restricted up to $20\%$ of this amount for the additional control approach. So, up to 118 kilos were spread over five additional control cycles, totaling approximately 24 kilos or up to $4\%$ per cycle ($0\leqslant u_{A}^{i} \leqslant 0.04; ~i = 1, 2, \ldots ,5$). For the adult phase, we divided the $20\%$ additional control into up to $10\%$ perifocal control and up to $10\%$ ULV spraying. In the case of perifocal control, we restricted up to $2\%$ or 0.43 l for additional control in each of the five cycles ($0 \leqslant u_{F_1}^{i} \leqslant 0.02; ~i = 1, 2, \ldots ,5$). Finally, for ULV spraying, we restricted up to 5 l or $5\%$ for additional control in each of the two cycles ($0 \leqslant u_{F_2}^{i} \leqslant 0.05; ~i = 1, 2$). Therefore, the control decision variables’ upper bounds in Eq. (14) were chosen to respect the threshold presented in Fig. 3.

### Fitting method

To fit the proposed epidemiological model, we used the lsqcurvefit function of MATLAB®  software. The goal is to optimize the recovery rate values of dengue virus for symptomatic ($\theta _I$) and asymptomatic ($\theta _M$) infected humans. With this, the evolution of the populations of humans and mosquitoes generated by the numerical simulation of the model Eq. (1) reproduces as faithfully as possible the reality of the populations of humans infected in our case study in the city of Belo Horizonte. Therefore, we also use the set of values of its parameters from Table 1 respecting Belo Horizonte’s climate. We used 2019, as the last dengue epidemic occurred, as the base year for control actions.

For the fit to be done, the lsqcurvefit function requires some values and their respective lower and upper limits to be supplied as input parameters. Based on the literature, we set the initial conditions of the parameters to be optimized. In this study, they are the humans’ recovery $\theta _I$ and $\theta _M$. The initial conditions of the state variables must also be informed, which were defined here based on information on the number of real cases of infected humans.

Particular attention must be given to the initial condition and limits established for susceptible and recovered humans. From the 116,320 confirmed cases of dengue registered for a population of 2.5 million in 2019, we verified that the incidence of cases was 4.65% of the population. Therefore, the number of 6% of the population, i.e., 150,000 susceptible, was considered reasonable, trying to include also cases of infected people who were not notified, assuming that they did not seek medical help, especially for asymptomatic people. Thus, analyzing the numbers of susceptible and recovered individuals, it can be seen that they make practical sense since the approximately 2.5 million city inhabitants already had the possibility of contact with dengue serotypes for many years. Furthermore, if a serotype infected them in other years, they became immune (recovered) to that serotype.

Information on the relationship between the number of humans and *Ae. aegypti* mosquitoes is still being determined in the literature. For example, Rodrigues et al.^28^ empirically estimated six adult females per human and three larvae per human. In this work, the assumption is that there must be at least one mosquito and up to five for each susceptible. The same was considered for the immature phase of the vector; therefore, the initial condition of 150 thousand was limited to 12.5 million immature and susceptibles adult vector forms. The initial condition was zero for exposed and infected mosquitoes, assuming the absence of dengue virus circulation. In^54^, a survey was conducted where 0.12% of the total number of mosquitoes captured were positive for the dengue virus. So, to also consider the percentage of exposed mosquitoes, we assumed up to 4% of the total susceptibles mosquitoes (i.e., 500,000) as an upper bound for exposed and infected mosquitoes. As for exposed humans, the initial condition is 1000, and the upper bound was considered to be 50,000, dividing the 150,000 of the initial susceptible condition between exposed, symptomatic infected, and asymptomatic infected populations.

Finally, for the fit, we also considered data on precipitation, temperature, and humidity for 2019 in Belo Horizonte. The study horizon contains 364 days, ignoring December 31, based on the 52 epidemiological weeks that a regular year has.

### Ethical approval

The Belo Horizonte City Hall and the CEFET-MG ethics committees approved this study to access information on how the *Ae. aegypti* mosquito is controlled in Belo Horizonte. Collecting personal information and participation authorization from city residents was not necessary.

## Results

### Model fitting

Table 4 has the initial condition values and their range, in addition to the optimal values found for the model fit.

**Table 4:** Model fit results for the year 2019.

	Initial condition	Lower bound	Upper bound	Optimal value
State variable				
*S*	150,000	90,000	2,500,000	149,629
*E*	1000	0	50,000	130
*M*	0	0	50,000	2917
*I*	0	0	50,000	7
*R*	2,350,000	2,350,000	2,500,000	2,350,000
*A*	150,000	0	12,500,000	195,632
$F_S$	150,000	0	12,500,000	618,490
$F_E$	0	0	500,000	27
$F_I$	0	0	500,000	0
Model parameter				
$\theta _M$	0.6324	0.0619	0.319	0.174
$\theta _I$	0.0975	0.01	0.50	0.3513

**Figure 6:**
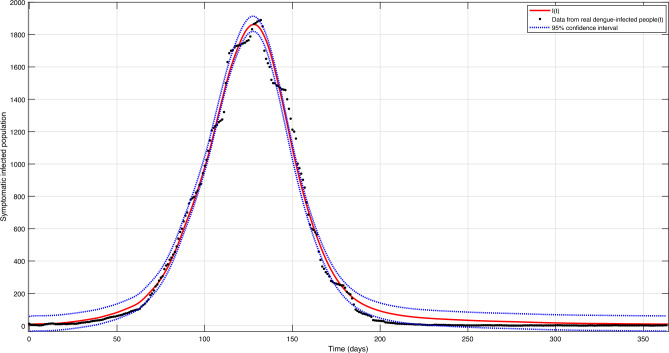
Curve fitting of the symptomatic infected population (*I*) from Belo Horizonte.

Figure 6 shows the fit of the simulated symptomatic infected humans to the real curve of confirmed infected. Moreover, Fig. 6 shows the 95% confidence interval for the mean difference between the number of infected estimated by the fitting method and the real infected, which is [− 42.52, 50.95].

**Figure 7:**
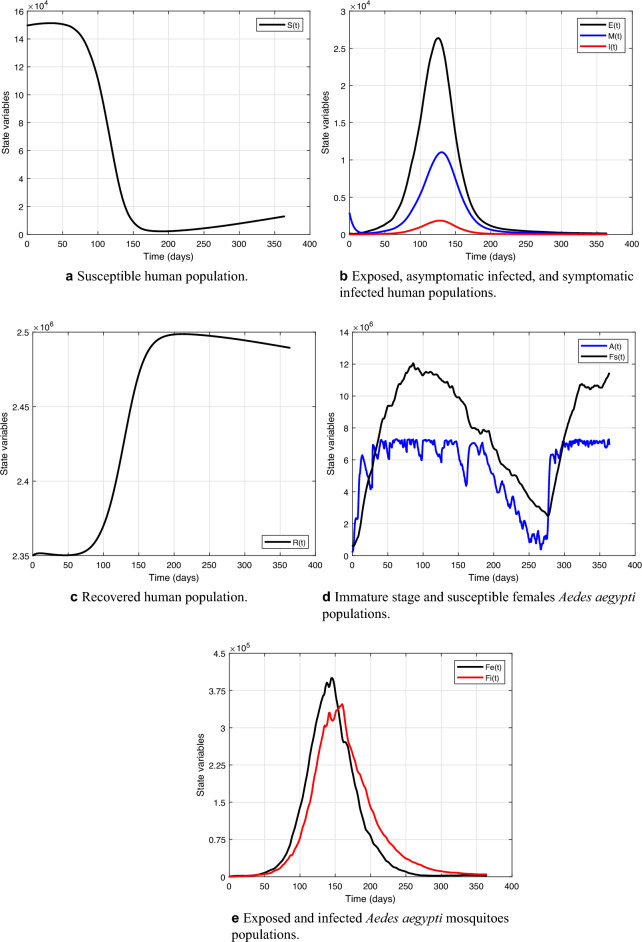
Populations evolution after model fitting for the year 2019.

Note how good the fit results were, getting very close to the real curve. Analyzing the fit results for the year 2019, the integral of the curve of the real infected is 116,320, and that of the infected simulated is 121,922, with the root mean squared error equal to 45.4. Figure 7 shows the evolution of human and mosquito populations after curve fitting, representing the final result of the mathematical model. It is essential to mention that in this fit, the control actions carried out in Belo Horizonte are already being considered. However, because of this high number of infected people, this situation gives rise to thinking about the need for additional control, as discussed earlier.

### Multiobjective results

**Figure 8:**
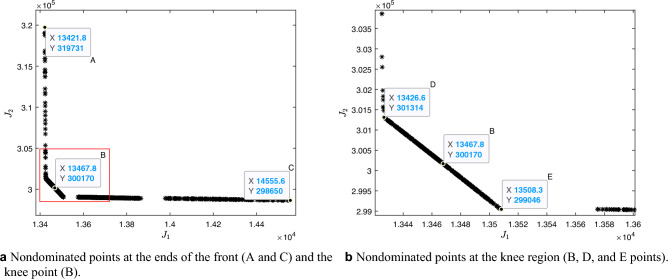
Nondominated front in the objectives space $J_1$ (the control costs) $\times J_2$ (the hospital costs). The figure on the right side (**b**) is a zoom of the knee region of the figure on the left side (**a**), selected in red.

After the 30 runs of the NSGA-II, Fig. 8a shows the final Pareto-optimal front. It is composed of 840 combined nondominated points found in all algorithmic runs. Information on the decision variable space and the combined result of all Pareto fronts found in the objective space can be found in the Supplementary Information.

The Pareto-optimal front has three well-defined regions. First, an almost horizontal region, close to the $J_1$ function axis; second, an almost vertical region, close to the $J_2$ function axis; and a third proportional region between the two previous regions, which corresponds to the “knee” of a curve shown in Fig. 8b. Therefore, to enhance comprehension of the obtained combined nondominated front, consider the five highlighted points in Fig. 8.

During the process of choosing these five points, we first select the two extremities points of the Pareto-optimal front, that is, where there is the highest hospital cost versus the lowest cost with additional control (point A) and where there is the lowest hospital cost versus the highest cost with additional control (point C). The other points chosen depict options that apparently have a lower hospital cost for an amount of control similar to that of point A and lower cost with control for a hospital cost similar to that of point B. Thus, of the five highlighted points, three (D, B, and E) belong to the knee region highlighted in red, and two (A and C) correspond to the extremes of the other identified regions.

For each of the five chosen points, the control strategies are different. It is up to the decision-maker to choose the strategy best suits its planning. Table 5 shows the percentage of additional control according to each point.

**Table 5:** Additional control percentage.

Point	$u_A$ (%)	$u_{F_1}$ (%)	$u_{F_2}$ (%)
A	5	5	2
B	5	10	6
C	14	10	10
D	5	10	2
E	5	10	10

To better understand the costs of control and hospital treatment of those infected at each point, check Table 6, which uses the costs for 2019 as a reference. Consider $J_1 + J_2 = J$ and the efficiency as the reduction of *J* value. A tiny observation is that the values appearing in the selected points’ labels in Fig. 8 are approximations since the software that generates the graphs rounds the values.

**Table 6:** Objective function value for the selected nondominated front points.

Point	$$J_1$$	$$J_2$$	*J*	Efficiency$$_J$$ (%)
A	US$ 13,421.83	US$ 319,731.33	US$ 333,153.16	2.14
B	US$ 13,467.27	US$ 300,186.55	US$ 313,653.82	7.87
C	US$ 14,555.55	US$ 298,649.59	**US$ 313,205.14**	8
D	US$ 13,426.60	US$ 301,313.64	US$ 314,740.24	7.55
E	US$ 13,508.34	US$ 299,046.36	**US$ 312,554.70**	8.19

Significant values are in [bold].

From the point of view of the efficiency of the objective function value, points E, C and B, in that order, showed the most significant reduction after the additional control compared to the objective function value without additional control. Points B and E are similar in the amount of additional control. One issue that draws attention is the comparison between points A and D. The difference between them is a modest amount of additional perifocal control, showing that this small amount brings a considerable response in the objective function. Given the perifocal control effectiveness, the hospital cost is significantly reduced and impacts the value of *J*. In this sense, between A and D, the most coherent choice for the decision-maker would be point D, located near the knee region.

**Figure 9:**
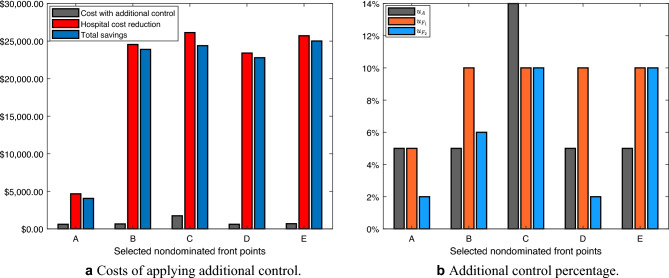
Different percentages of larvicide and adulticide and their associated decrease in hospital costs, increase in vector control costs, and changes in total costs for the selected nondominated front points.

Figure 9a shows how much could be saved by implementing additional control measures. It also shows reduced hospital costs due to decreased symptomatic infected people. As expected, the total savings are higher for points C and E, at US$ 24,388.72 and US$ 25,018.03, respectively. The difference between the total savings of these points is that in C, more was spent on additional control than in E, but also resulting in more significant hospital cost reduction.

To carry out the sensitivity analysis of the values obtained as total savings, we considered an experiment in which, using the K-nearest neighbors (KNN) method, we found the 10 points closest to each of the five previously selected points (A, B, C, D, and E). Then, we evaluated the decision variables in the dynamic system for each point and obtained the following confidence intervals. Point A-CI: [US$ 4,769.96; US$ 9,606.81]; point B-CI: [US$ 23,887.71; US$ 23,966.31]; point C-CI: [US$ 24,411.42; US$ 24,452.42]; point D-CI: [US$ 22,342.37; US$ 22,903.51]; point E-CI: [US$ 24,984.28; US$ 25,055.79].

Figure 9b shows the percentage of additional control associated with each selected nondominated point. There is a discrepancy regarding the amount of larvicide applied between points C and E. In fact, Fig. 9 shows that the more significant amount found in C further reduces hospital costs but makes the objective function more expensive. In general, among all the points, the decision-maker would probably choose point E, which is closer to the knee of the curves and had the lowest cost of the operation.

To conclude the study of the differences between the selected nondominated points, consider Table 7, which reveals the reduction of the symptomatic dengue-infected after additional control actions in each of the five points tested. The efficiency of the reduction of infected was also calculated, consisting of the comparison with the total number of existing infected without additional control.

Concerning the efficiency of the objective function value, points C, E, and B, in that order, showed the most significant reduction after the additional control. In this case, the reduction in the number of infected people who would be hospitalized without additional control actions is more evident at point C, with 156 fewer infected than at point E.

**Table 7:** Reduction of symptomatic infections for the selected nondominated front points.

Point	Infected without additional control	Infected with additional control	Reduction of infected people	Efficiency$_I$ (%)
A	121,922	120,175	1747	1.43
B		112,777	9145	7.5
C		112,187	**9735**	7.98
D		113,203	8719	7.15
E		112,343	**9579**	7.86

Significant values are in [bold].

Therefore, the decision maker would probably choose a reduced number of infections among the options presented, which corresponds to the point C control approach. Due to the trade-off nature of the multiobjective problem, it is clear that the reduction of infected is more significant than in point E, although the cost of the additional control operation is slightly higher. However, if there are few financial resources for additional control, the decision maker could choose point E, which would also reduce the number of infected people, but less than point C.

**Figure 10:**
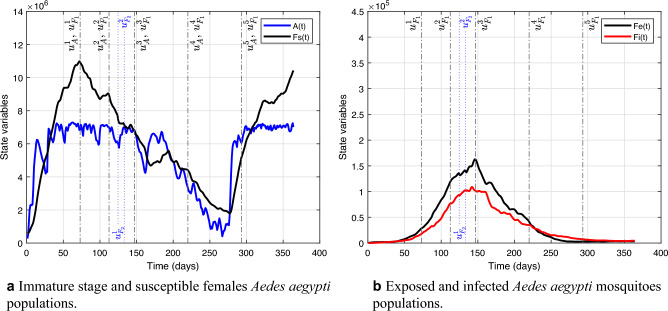
Mosquito populations evolution after point C additional control measure implementation. The vertical lines represent the implementation start dates for each additional control cycle.

Figure 10 shows the estimated temporal dynamics of the mosquito populations after point C interventions to evaluate the impact of this best control measure on the mosquito populations. We added vertical lines to represent the start dates of the implementation of each intervention. So, Fig. 10a shows the five additional larvicide control cycles in the immature phase, the five additional perifocal control cycles, and the two additional ULV spraying cycles in the adult phase of susceptibles mosquitoes. Figure 10b shows the five additional perifocal control cycles and the two additional ULV spraying cycles in the adult phase of exposed and infected mosquitoes. In comparison with Fig. 7d,e, we can see that the most significant reduction was in the exposed and infected mosquitoes population.

**Figure 11:**
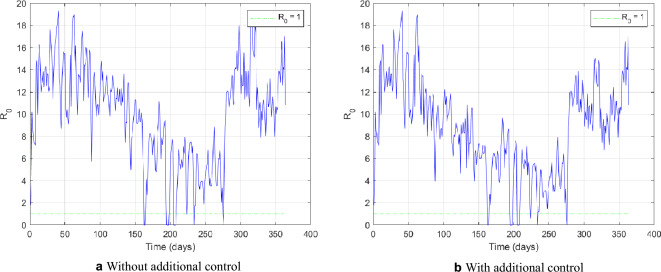
Evolution of the threshold $R_0$ without and with additional control actions considering the measures defined in point C.

The analyzed additional interventions still need to be increased to keep the basic reproduction number below the critical threshold, as shown in Fig. 11 after point C additional control measure implementation. Soon after the end of the residual effect of the control, the system converges to the equilibrium point $P_{1}$, an asymptotically stable node when there is an epidemic to be controlled. The Belo Horizonte City Hall should take more actions to prevent an epidemic, such as a significant number of additional control cycles and effective actions to raise public awareness about removing potential breeding sites. However, the latter is beyond the scope of this work.

## Discussion

The nature of the spread of dengue is complex. Understanding it and replicating it in a mathematical model, aiming to obtain predictive information, is a task that involves several fields of knowledge. It is necessary to observe the climatic influence and its variations in precipitation, temperature, and humidity, which as a rule, can change and, consequently, modify the *Ae. aegypti* life cycle. It is necessary to understand the dynamics between the vector and humans since the mosquito lives in the home environment. It is also necessary to verify whether the decision-making of health managers is being carried out and, if so, with what quality, or even find out if new control measures can or should, be carried out. Many other variables could be mentioned here to reinforce the idea that solving real-world problems is challenging. However, through multiobjective optimization, this work promoted the simultaneous evaluation of some complex variables. Thus, we were able to promote a useful mathematical and computational framework for health managers to the point of generating simplified results and information.

For this, we proposed a new epidemiological mathematical model—with emphasis on incorporating asymptomatic populations, which accelerate the dissemination of the dengue virus, and on the aquatic phase of the vector—so that it would be possible to simulate the application of larvicides, as it occurs by the recommendations of the Ministry of Health to face the vector. Some model parameters are influenced by climatic variables directly related to the mosquito life cycle. The model, being generic, can be applied to any city. In this work, we carried out a case study for the city of Belo Horizonte, Minas Gerais, Brazil, and therefore, we used the model with real data to represent the curve of infected people in the city during the epidemic years.

Next, we presented the multiobjective optimization results using the NSGA-II genetic algorithm. A significant contribution of this work to the literature and society is to have presented the trade-off between the costs involving additional vector control versus the reduced need for public hospital treatment. The additional control approach we proposed was cost-effective from a financial point of view; however, the most relevant finding was the decrease in infected people and the need for hospital treatment compared to the approach without additional control.

In this sense, the health manager will be equipped with information for better decision-making. From now on, lessons learned from the epidemic years can be used to prepare control actions and support awareness campaigns about the risks of dengue (both in the media and in schools). Thus, the population will gain in terms of health, especially the less favored. The lower the need for hospitalization, the lower the exposure to biological risks that can lead to complications in the clinical condition of patients.

During other epidemic years, the health manager can monitor the number of identified dengue cases and then use our methodology to adjust the model to this new scenario. Depending on their needs, the health manager can use smaller or larger time windows than the 1-year period we tested for the 2019 case study. In this way, adopting the estimated optimal control measures and knowing the best moment for implementing the interventions is possible. There is also the mosquito abundance indicator Larval Index Rapid Assay for Aedes aegypti (LIRAa) for entomological surveillance of Aedes, widely used in Brazil by the Ministry of Health. Monitoring this indicator could be coupled in future works to act more effectively in control actions to avoid the beginning of an epidemic.

Limitations of the case study include underreporting of symptomatic infected data and the need for more information on the ideal number of additional control cycles, especially for ULV spraying. When evaluating the effort to apply additional control by Belo Horizonte City Hall, some points were not taken into account, e.g., personnel costs (hiring or overtime and uniforms for endemic diseases combat agents), as well as the cost of machinery, all covered by the City Hall.

There will also be productivity costs associated with people missing paid and unpaid work when they are ill, in treatment, or in premature death. We did not evaluate it in this work, but the additional vector control might save the government more money than just reduced public hospital admission. In Brazil, citizens’ public hospitalizations are free of charge and funded by the government. So, another possibility would be to assess dengue’s total economic burden by also including information on private hospitalizations, which in Brazil can be funded by companies when they pay for a private health insurance for their employees or by citizens themselves.

Hence, there is no doubt that this work opens up possibilities for solving future problems. This case study only considers insecticide vector control; however, in the future the model could be adapted to include other interventions against the spread of dengue fever, such as Wolbachia bacteria or vaccination. The effect of delayed oviposition and sterile males could also be included. Within the scope of the control application, other approaches could be tested and compared. As dengue is a multifactorial disease, control approaches must be multiple and ecologically structured. In this sense, it is worth exploring the possibility of including the risk of systematic insecticide use and possible environmental impacts in the model. Complementary measures to combat the vector, e.g., intensifying campaigns to raise awareness of the population on mechanical control would also be appropriate. Finally, another interesting approach would be to propose computational models based on cellular automata.

In summary, we develop in this work a novel epidemiological mathematical model and a solution to a multiobjective problem for which we show a set of compromise solutions between the costs of controlling the *Ae. aegypti* and the number of dengue-infected people considering real data in the case study for the city of Belo Horizonte, which decision-makers can use as a range of options to implement a successful control action according to the financial and social condition of the case study city.

## Supplementary Information

Supplementary Information.

## Data Availability

The datasets analyzed during the current study are available in the public databases, such as The DATASUS (Department of Informatics of the Brazilian National Health System—SUS^51,53^) for dengue case data, and the National Institute of Meteorology (INMET^50^), for daily weather data of total precipitation, average temperature, and average humidity for the city of Belo Horizonte. Information on the type and amount of control already employed in Belo Horizonte was made available by its City Hall through access to information law.
