# Supplementary material for: Multiobjective optimization to assess dengue control costs using a climate-dependent epidemiological model
Source: Sci Rep. 2023 Jun 24;13:10271. doi: 10.1038/s41598-023-36903-w (PMC10290689; doi:10.1038/s41598-023-36903-w)
Supplement: Supplementary file 1 — Supplementary Information. [file 41598_2023_36903_MOESM1_ESM.pdf]

Supplementary Information for Multiobjective optimization to assess dengue control costs using a climate-dependent epidemiological model

Amália Soares Vieira de Vasconcelos[1,*], Josenildo Silva de Lima[1], and Rodrigo Tomás Nogueira Cardoso[2]

[1]Postgraduate Program in Mathematical and Computational Modeling (PPGMMC), Federal Center for Technological Education - CEFET-MG, Av. Amazonas, 7675, Nova Gameleira, Belo Horizonte 30510-000, Minas Gerais, Brazil
[2]Department of Mathematics, Federal Center for Technological Education - CEFET-MG, Av. Amazonas, 7675, Nova Gameleira, Belo Horizonte 30510-000, Minas Gerais, Brazil
[*]amaliasv@hotmail.com

Materials and methods

The model

In this work, a new epidemiological mathematical model is proposed to represent the populations of the vector *Ae. aegypti*, human populations, and the interactions between these populations for the spread of dengue. First, consider the nine state variables depicted below:

State variable	Descriptions
S	Number of humans susceptible to the dengue virus
E	Number of humans exposed to the dengue virus
M	Number of asymptomatic susceptible humans infected with the dengue virus
I	Number of symptomatic susceptible humans infected with the dengue virus
R	Number of humans recovered immune to the dengue virus
A	Mosquito population in the immature stage
F_S	The population of susceptible adult females
F_E	The population of exposed adult females
F_I	The population of infected adult females

Finally, the model is formalized mathematically by the following ordinary differential equations:

$$\begin{cases} \dfrac{dS}{dt} = & \mu N - \dfrac{\xi \psi S F_I}{N} - \mu S \\ \dfrac{dE}{dt} = & \dfrac{\xi \psi S F_I}{N} - (\nu + \mu) E \\ \dfrac{dM}{dt} = & (1-\eta)\nu E - (\theta_M + \mu) M \\ \dfrac{dI}{dt} = & \eta \nu E - (\theta_I + \mu) I \\ \dfrac{dR}{dt} = & \theta_M M + \theta_I I - \mu R \\ \dfrac{dA}{dt} = & \varepsilon \phi \left(1 - \dfrac{A}{C}\right) F - (\alpha + \mu_A + u_A) A \\ \dfrac{dF_S}{dt} = & \sigma \alpha A - (\mu_F + u_F) F_S - \dfrac{\beta \xi F_S (M+I)}{N} \\ \dfrac{dF_E}{dt} = & \dfrac{\beta \xi F_S (M+I)}{N} - (\gamma + \mu_F + u_F) F_E \\ \dfrac{dF_I}{dt} = & \gamma F_E - (\mu_F + u_F) F_I, \end{cases} \tag{1}$$

in which, $\mu, N, \xi, \psi, \eta, \nu, \varepsilon, \theta_M, \theta_I, \phi, C, \gamma, \sigma, \alpha, \beta, \mu_A, \mu_F, u_A, u_F \in \mathbb{R}^+$ and $F_S + F_E + F_I = F$. The diagram of the dynamics of interactions between human and mosquito populations can be seen in Figure S1.

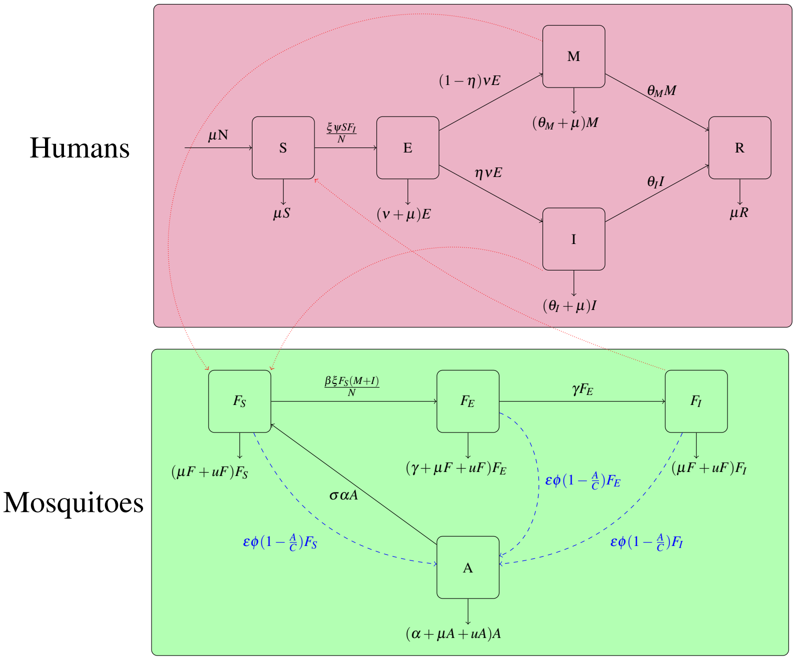

Figure S1. Epidemiological model diagram. The red dotted line (⋯) represents interactions between human and mosquito populations. The blue dashed line (- - -) shows the interactions between the immature and adult stages of the vector *Aedes aegypti*.

The equilibrium points

The regions of the system Equation (1) with biological sense are defined by:

$$\Gamma_1 = \{(A, F_S, F_E, F_I) \in \mathbb{R}^4 \mid A, F \geqslant 0 \text{ and } 0 \leqslant A \leqslant C\} \quad \text{and} \quad \Gamma_2 = \{(S, E, M, I, R) \in \mathbb{R}^5 \mid 0 < S, E, M, I, R < N\}, \tag{2}$$

that is, the populations are nonnegative, the immature stage population A does not exceed the carrying capacity C, and the human populations do not exceed the total number N of humans. Considering the values of the epidemiological parameters of this system invariant in time and assuming the existence of the mosquito population, two equilibrium points can be determined, given by:

1) $P_0 = (N, 0, 0, 0, 0, \bar{A}, \bar{F_S}, 0, 0)$, which is the trivial or disease-free equilibrium point, $\bar{A} = C\left(1 - \frac{1}{Q_0}\right)$ and $\bar{F_S} = \frac{\sigma\alpha}{\mu_F + u_F}\bar{A}$. In this case, both the mosquito and human populations are free of the dengue virus.

2) $P_1 = (S^{**}, E^{**}, M^{**}, I^{**}, R^{**}, A^{**}, F_S^{**}, F_E^{**}, F_I^{**})$, which is the nontrivial equilibrium or epidemic equilibrium point, *i.e.*, when the dengue virus is present. In this case,

$$S^{**} = \frac{\mu N}{\xi\psi\frac{F_I^{**}}{N} + \mu}, \qquad E^{**} = \frac{\xi\psi F_I^{**}\mu N(\nu+\mu)}{\xi\psi F_I^{**} + \mu N}, \qquad M^{**} = \frac{(1-\eta)\nu\xi\psi F_I^{**}\mu N(\nu+\mu)}{(\theta_M+\mu)(\xi\psi F_I^{**} + \mu N)},$$

$$I^{**} = \frac{\eta\nu\xi\psi F_I^{**}\mu N(\nu+\mu)}{(\theta_I+\mu)(\xi\psi F_I^{**} + \mu N)}, \qquad R^{**} = \frac{\theta_M M^{**} + \theta_I I^{**}}{\mu}, \qquad A^{**} = C - \frac{C(\alpha+\mu_A+u_A)(\mu_F+u_F)}{\sigma\alpha\varepsilon\phi},$$

$$F_S^{**} = \frac{\sigma\alpha C - \left[\frac{C(\alpha+\mu_A+u_A)(\mu_F+u_F)}{\varepsilon\phi}\right]}{\left[(\mu_F+u_F) + \frac{\beta\xi(M+I)}{N}\right]}, \qquad F_E^{**} = \frac{\beta\xi F_S^{**}(M+I)}{N(\gamma+\mu_F+u_F)}, \quad \text{and} \quad F_I^{**} = \frac{\gamma F_E^{**}}{N(\mu_F+u_F)}.$$

During establishing the model's trivial equilibrium point, two thresholds are found: the basic offspring number (Q_0) and the basic reproduction number (R_0). Both were obtained using the next-generation matrix method[1], as explained below.

The basic offspring number

As the interest is in the vector and there is no dengue virus circulation, then the system Equation (1) can be rewritten as:

$$\begin{cases} \dfrac{dA}{dt} &= \varepsilon\phi\left(1-\dfrac{A}{C}\right)F-(\alpha+\mu_A+u_A)A \\ \\ \dfrac{dF}{dt} &= \sigma\alpha A-(\mu_F+u_F)F. \end{cases} \tag{3}$$

Defining x as the set of all model variables, then $x_1 = A$ and $x_2 = F$. Thus, the system Equation 3 can be rewritten as:

$$\frac{dx_i}{dt} = V_i(x) - W_i(x), \tag{4}$$

in which $V_i(x)$ is the deposition of eggs in each population i, with $i = 1,2$, and $W_i = W_i^- - W_i^+$, where W_i^+ is the rate of transfer of female mosquitoes into a compartment and W_i^- is the rate of transfer of female mosquitoes out of the compartment. In this case, V and W are defined by:

$$V = \begin{bmatrix} \varepsilon\phi\left(1-\dfrac{A}{C}\right)F \\ \\ 0 \end{bmatrix} \text{ and } W = \begin{bmatrix} (\alpha+\mu_A+u_A)A \\ \\ (\mu_F+u_F)F-\sigma\alpha A \end{bmatrix}.$$

The Jacobian matrices of birth, with $\mathscr{V}_{ij} = \frac{\partial V_i}{\partial x_j}\Big|_{P_0}$, and of transition, with $\mathscr{W}_{ij} = \frac{\partial W_i}{\partial x_j}\Big|_{P_0}$, evaluated at the trivial equilibrium point P_0, are:

$$\mathscr{V} = \begin{bmatrix} 0 & \varepsilon\phi \\ \\ 0 & 0 \end{bmatrix} \text{ and } \mathscr{W} = \begin{bmatrix} (\alpha+\mu_A+u_A) & 0 \\ \\ -\sigma\alpha & (\mu_F+u_F) \end{bmatrix}.$$

The matrix $\mathscr{W}^{-1}$ is:

$$\mathscr{W}^{-1} = \begin{bmatrix} \dfrac{1}{(\alpha+\mu_A+u_A)} & 0 \\ \\ \dfrac{\sigma\alpha}{(\alpha+\mu_A+u_A)(\mu_F+u_F)} & \dfrac{1}{(\mu_F+u_F)} \end{bmatrix}.$$

Finally, the next-generation matrix of the system is given by $\mathscr{V}\mathscr{W}^{-1}$:

$$\mathscr{V}\mathscr{W}^{-1} = \begin{bmatrix} \dfrac{\varepsilon\phi\sigma\alpha}{(\alpha+\mu_A+u_A)(\mu_F+u_F)} & \dfrac{\varepsilon\phi}{(\mu_F+u_F)} \\ \\ 0 & 0 \end{bmatrix}.$$

The basic offspring number is defined as the spectral radius of the next generation operator, being the largest eigenvalue in modulus of $\rho(\mathscr{V}\mathscr{W}^{-1})$. Equation (5) describes Q_0:

$$Q_0 = \rho(\mathscr{V}\mathscr{W}^{-1}) = \frac{\sigma\alpha}{(\alpha+\mu_A+u_A)}\frac{\varepsilon\phi}{(\mu_F+u_F)}. \tag{5}$$

Notice that Q_0 does not depend on the carrying capacity C and that $Q_0 > 0$ because all model parameters are positive.

The basic reproduction number

The basic offspring number, derived from demography and ecology, inspired epidemiology to establish what is now known as the reproduction number, R_0[2]. It is the same idea: measuring the number of secondary infections from an infected individual in a susceptible population[3]. Therefore, the basic reproduction number is a threshold of extreme relevance in studying infectious diseases, as it will be possible to infer the power of transmission of these diseases.

In this sense, R_0 is also essential to infer about[2] control strategies. When $R_0 < 1$, an infected person produces on average less than a new infected person. Therefore, the infection, which causes a particular disease, may disappear if infection rates are constant. However, when $R_0 > 1$, the disease spreads quickly and can even generate an epidemic if the value is high.

Thus, the interest is to evaluate the compartments that are infectious terms of the model Equation (1), that is, E, M, I, F_E and F_I. Again, the next-generation matrix method[1] was used to find R_0 according to the expression:

$$R_0 = \rho(\mathscr{K}\mathscr{T}^{-1}), \tag{6}$$

in which $\mathscr{K}$ is the Jacobian matrix that describes the production of new infected and $\mathscr{T}$ is the Jacobian matrix that describes the state changes. Thus, the following matrices are produced:

$$K = \begin{bmatrix} \dfrac{\xi\psi S F_I}{N} \\ 0 \\ 0 \\ \dfrac{\beta\xi F_S(M+I)}{N} \\ 0 \end{bmatrix} \text{ and } T = \begin{bmatrix} (\nu+\mu)E \\ -(1-\eta)\nu E + (\theta_M+\mu)M \\ -\eta\nu E + (\theta_I+\mu)I \\ (\gamma+\mu_F+u_F)F_E \\ -\gamma F_E + (\mu_F+u_F)F_I \end{bmatrix}. \tag{7}$$

To obtain the next-generation operator, $\mathscr{K}\mathscr{T}^{-1}$, calculate $\mathscr{K}_{ij} = \frac{\partial K_i}{\partial x_j}|_{P_0}$ and $\mathscr{T}_{ij} = \frac{\partial T_i}{\partial x_j}|_{P_0}$. Then, the Jacobian matrices of the infection terms $\mathscr{K}$ and the transition terms $\mathscr{T}$ evaluated at the disease-free equilibrium point are defined by:

$$\mathscr{K} = \begin{bmatrix} 0 & 0 & 0 & 0 & \xi\psi \\ 0 & 0 & 0 & 0 & 0 \\ 0 & 0 & 0 & 0 & 0 \\ 0 & \dfrac{\beta\xi\bar{F}_S}{N} & \dfrac{\beta\xi\bar{F}_S}{N} & 0 & 0 \\ 0 & 0 & 0 & 0 & 0 \end{bmatrix}$$

and

$$\mathscr{T} = \begin{bmatrix} \nu+\mu & 0 & 0 & 0 & 0 \\ -(1-\eta)\nu & \theta_M+\mu & 0 & 0 & 0 \\ -\eta\nu & 0 & \theta_I+\mu & 0 & 0 \\ 0 & 0 & 0 & \gamma+\mu_F+u_F & 0 \\ 0 & 0 & 0 & -\gamma & \mu_F+u_F \end{bmatrix}.$$

The matrix $\mathscr{T}^{-1}$ is:

$$\mathscr{T}^{-1} = \begin{bmatrix} \dfrac{1}{\nu+\mu} & 0 & 0 & 0 & 0 \\ -\dfrac{(\eta-1)\nu}{(\nu+\mu)(\theta_M+\mu)} & \dfrac{1}{\theta_M+\mu} & 0 & 0 & 0 \\ \dfrac{\eta\nu}{(\nu+\mu)(\theta_I+\mu)} & 0 & \dfrac{1}{\theta_I+\mu} & 0 & 0 \\ 0 & 0 & 0 & \dfrac{1}{\gamma+\mu_F+u_F} & 0 \\ 0 & 0 & 0 & \dfrac{\gamma}{(\gamma+\mu_F+u_F)(\mu_F+u_F)} & \dfrac{1}{\mu_F+u_F} \end{bmatrix}.$$

The next generation matrix of the system is given by $\mathscr{K}\mathscr{T}^{-1}$:

$$\mathscr{K}\mathscr{T}^{-1} = \begin{bmatrix} 0 & 0 & 0 & \frac{\gamma\xi\psi}{(\gamma+\mu_F+u_F)(\mu_F+u_F)} & \frac{\xi\psi}{(\mu_F+u_F)} \\ 0 & 0 & 0 & 0 & 0 \\ 0 & 0 & 0 & 0 & 0 \\ \frac{\beta\xi\bar{F_S}\eta\nu}{(\nu+\mu)(\theta_I+\mu)N} & -\frac{\beta\xi\bar{F_S}(\eta-1)\nu}{(\nu+\mu)(\theta_M+\mu)N} & \frac{\beta\xi\bar{F_S}}{(\theta_M+\mu)N} & \frac{\beta\xi\bar{F_S}}{(\theta_I+\mu)N} & 0 & 0 \\ 0 & 0 & 0 & 0 & 0 \end{bmatrix}.$$

The eigenvalues of the matrix $\mathscr{K}\mathscr{T}^{-1}$ are given by the roots of the characteristic equation $p(\lambda) = \det(\lambda I - \mathscr{K}\mathscr{T}^{-1})$. So, the characteristic polynomial is:

$$-\lambda^3\left[-\lambda^2 + \frac{\gamma\xi\psi}{(\gamma+\mu_F+u_F)(\mu_F+u_F)} \times \left(\frac{\beta\xi\bar{F_S}\eta\nu}{(\nu+\mu)(\theta_I+\mu)N} - \frac{\beta\xi\bar{F_S}(\eta-1)\nu}{(\nu+\mu)(\theta_M+\mu)N}\right)\right] = 0. \tag{8}$$

Thus, from the model Equation (1), the expression for R_0 is obtained as the largest eigenvalue in modulus:

$$R_0 = \frac{\gamma\xi\psi}{(\gamma+\mu_F+u_F)(\mu_F+u_F)} \times \left[\frac{\beta\xi\bar{F_S}\eta\nu}{(\nu+\mu)(\theta_I+\mu)N} + \frac{\beta\xi\bar{F_S}(\eta-1)\nu}{(\nu+\mu)(\theta_M+\mu)N}\right] \tag{9}$$

The threshold R_0 can be interpreted biologically as the product between human and mosquito infectious terms. Note that in R_0, there is a dependence on important mosquito and human parameters, also between susceptible mosquitoes and the size of the total human population. Because R_0 depends on $\bar{F_S}$, which, in turn, depends on Q_0, this can be seen as a risk factor for dengue outbreaks.

Suppose a single infected female is introduced in a completely susceptible population of mosquitoes and humans. The first term of R_0, given by $\frac{\gamma\xi\psi}{(\gamma+\mu_F+u_F)(\mu_F+u_F)}$, shows that this single female will bite humans and transmit the dengue virus, given the probability that exposed mosquitoes survive the extrinsic incubation period and become infectious.

Now suppose the introduction of a single infected human in a completely susceptible population of mosquitoes and humans. The dynamics would follow a pattern similar to that mentioned above. In the case of the second term of R_0, which is $\frac{\beta\xi\bar{F_S}\eta\nu}{(\nu+\mu)(\theta_I+\mu)N}$, the interpretation is that the infected human would survive the intrinsic incubation and the symptoms of the disease, and there may be a transmission of the dengue virus from that human to mosquitoes, given the probability of transmission and the total number of daily bites.

For the third term of R_0, $\frac{\beta\xi\bar{F_S}(\eta-1)\nu}{(\nu+\mu)(\theta_M+ mu)N}$, the difference concerning the second term is that the human would be infected but would not show symptoms. In any case, symptomatic and asymptomatic infected individuals are quite evident in R_0. According to Equation (9), control actions in the immature and adult stages attenuate the basic reproduction number, reducing the population of susceptible mosquitoes.

The stability analysis

For the stability analysis of the equilibrium points P_0 and P_1, consider the characteristic equation corresponding to the Jacobian matrix of the system Equation (1), given by:

$$J = \begin{bmatrix} -\frac{\xi\psi F_I}{N}-\mu & 0 & 0 & 0 & 0 & 0 & 0 & 0 & -\frac{\xi\psi S}{N} \\ \frac{\xi\psi F_I}{N} & -(\nu+\mu) & 0 & 0 & 0 & 0 & 0 & 0 & \frac{\xi\psi S}{N} \\ 0 & (1-\eta)\nu & -(\theta_M+\mu) & 0 & 0 & 0 & 0 & 0 & 0 \\ 0 & \eta\nu & 0 & -(\theta_I+\mu) & 0 & 0 & 0 & 0 & 0 \\ 0 & 0 & \theta_M & \theta_I & -\mu & 0 & 0 & 0 & 0 \\ 0 & 0 & 0 & 0 & 0 & -\frac{\varepsilon\phi F}{C}-(\alpha+\mu_A+u_A) & \frac{\varepsilon\phi(C-A)}{C} & \frac{\varepsilon\phi(C-A)}{C} & \frac{\varepsilon\phi(C-A)}{C} \\ 0 & 0 & -\frac{\beta\xi F_S}{N} & -\frac{\beta\xi F_S}{N} & 0 & \sigma\alpha & -(\mu_F+u_F)-\frac{\beta\xi(M+I)}{N} & 0 & 0 \\ 0 & 0 & \frac{\beta\xi F_S}{N} & \frac{\beta\xi F_S}{N} & 0 & 0 & \frac{\beta\xi(M+I)}{N} & -(\gamma+\mu_F+u_F) & 0 \\ 0 & 0 & 0 & 0 & 0 & 0 & 0 & \gamma & -(\mu_F+u_F) \end{bmatrix}. \tag{10}$$

According to[4], analyzing the stability of large systems is analytically unfeasible because of the calculations of all principal minors of the Jacobian matrix evaluated at the equilibrium points. So, one of the ways to assess stability is to use the Routh-Hurwitz criterion, which verifies the coefficients of the characteristic polynomial. In this sense,[4] prove that all Routh-Hurwitz conditions can be summarized by analyzing the term independent of the characteristic polynomial:

$$p(\lambda) = \det(J-\lambda I). \tag{11}$$

The independent term of the characteristic equation, designated by a_n, which is given by $a_n = a_n(0) = \det(J)$ is written as:

$$a_n = \frac{\beta\,\xi\,F_S\,\varepsilon\,\phi\,\mu\left(\frac{\xi\psi F_I}{N}+\mu\right)(\nu+\mu)(\theta_M+\mu)(\theta_I+\mu)\left(\frac{\varepsilon\phi F}{C}+\alpha+\mu_A+u_A\right)\left(\mu_F+u_F+\frac{\beta\xi(M+I)}{N}\right)(\gamma+\mu_F+u_F)(\mu_F+u_F)(A-C)}{CN}.$$

The local stability of the trivial equilibrium point

The Jacobian matrix of the system Equation (1) applied at the equilibrium point P_0 is:

$$J(P_0) = \begin{bmatrix} -\mu & 0 & 0 & 0 & 0 & 0 & 0 & 0 & -\xi\psi \\ 0 & -(\nu+\mu) & 0 & 0 & 0 & 0 & 0 & 0 & \xi\psi \\ 0 & (1-\eta)\nu & -(\theta_M+\mu) & 0 & 0 & 0 & 0 & 0 & 0 \\ 0 & \eta\nu & 0 & -(\theta_I+\mu) & 0 & 0 & 0 & 0 & 0 \\ 0 & 0 & \theta_M & \theta_I & -\mu & 0 & 0 & 0 & 0 \\ 0 & 0 & 0 & 0 & 0 & -\dfrac{\varepsilon\phi\bar{F}_S}{C} - (\alpha+\mu_A+u_A) & \dfrac{\varepsilon\phi(C-\bar{A})}{C} & \dfrac{\varepsilon\phi(C-\bar{A})}{C} & \dfrac{\varepsilon\phi(C-\bar{A})}{C} \\ 0 & 0 & -\dfrac{\beta\xi\bar{F}_S}{N} & -\dfrac{\beta\xi\bar{F}_S}{N} & 0 & \sigma\alpha & -(\mu_F+u_F) & 0 & 0 \\ 0 & 0 & \dfrac{\beta\xi\bar{F}_S}{N} & \dfrac{\beta\xi\bar{F}_S}{N} & 0 & 0 & 0 & -(\gamma+\mu_F+u_F) & 0 \\ 0 & 0 & 0 & 0 & 0 & 0 & 0 & \gamma & -(\mu_F+u_F) \end{bmatrix}.$$

Lemma 1 *The disease-free equilibrium point P_0 is locally asymptotically stable if the independent term a_n of λ of the polynomial given by the expression $\Lambda_n(\lambda) = \lambda^n + a_1\lambda^{n-1} + \cdots + a_{n-1}\lambda + a_n$ is strictly positive, and unstable if a_n is strictly negative*[4].

Proposition 1 ***(The local stability of the disease-free equilibrium point).*** *The disease-free equilibrium point P_0 is an asymptotically stable node if $0 < R_0 < 1$.*

Demonstration:

For the disease-free equilibrium point, applying P_0 in a_n is found

$$a_n = \frac{\beta\,\xi\,\bar{F}_S\,\varepsilon\,\phi\,\mu^2\,(\nu+\mu)\,(\theta_M+\mu)\,(\theta_I+\mu)\left(\dfrac{\varepsilon\phi(\bar{F}_S)}{C}+\alpha+\mu_A+u_A\right)(\mu_F+u_F)\,(\gamma+\mu_F+u_F)\,(\mu_F+u_F)\,\left(\bar{A}-C\right)}{CN}$$

Therefore, as a_n is positive, the disease-free equilibrium point P_0 is locally asymptotically stable for $0 < R_0 < 1$.

□

The local stability of the nontrivial equilibrium point

The Jacobian matrix of the system Equation (1) applied at the equilibrium point P_1 can be written as:

$$J(P_1)=\begin{bmatrix} J_1 & J_2 & J_3 \\ J_4 & J_5 & J_6 \\ J_7 & J_8 & J_9 \end{bmatrix}. \tag{12}$$

in which, J_i with $i=1,\ldots,9$ are the matrices 3×3 given below:

$$J_1=\begin{bmatrix} -\frac{\xi\psi F_I^{**}}{N}-\mu & 0 & 0 \\ \frac{\xi\psi F_I^{**}}{N} & -(\nu+\mu) & 0 \\ 0 & (1-\eta)\nu & -(\theta_M+\mu) \end{bmatrix},\ J_2=\begin{bmatrix} 0 & 0 & 0 \\ 0 & 0 & 0 \\ 0 & 0 & 0 \end{bmatrix},\ J_3=\begin{bmatrix} 0 & 0 & -\frac{\xi\psi S^{**}}{N} \\ 0 & 0 & \frac{\xi\psi S^{**}}{N} \\ 0 & 0 & 0 \end{bmatrix},$$

$$J_4=\begin{bmatrix} 0 & \eta\nu & 0 \\ 0 & 0 & \theta_M \\ 0 & 0 & 0 \end{bmatrix},\ J_5=\begin{bmatrix} -(\theta_I+\mu) & 0 & 0 \\ \theta_I & -\mu & 0 \\ 0 & 0 & -\frac{\varepsilon\phi F_S^{**}}{C}-(\alpha+\mu_A+u_A) \end{bmatrix},\ J_6=\begin{bmatrix} 0 & 0 & 0 \\ 0 & 0 & 0 \\ \frac{\varepsilon\phi(C-A^{**})}{C} & \frac{\varepsilon\phi(C-A^{**})}{C} & \frac{\varepsilon\phi(C-A^{**})}{C} \end{bmatrix},$$

$$J_7=\begin{bmatrix} 0 & 0 & -\frac{\beta\xi F_S^{**}}{N} \\ 0 & 0 & \frac{\beta\xi F_S^{**}}{N} \\ 0 & 0 & 0 \end{bmatrix},\ J_8=\begin{bmatrix} -\frac{\beta\xi F_S^{**}}{N} & 0 & \sigma\alpha \\ \frac{\beta\xi F_S^{**}}{N} & 0 & 0 \\ 0 & 0 & 0 \end{bmatrix}\text{ and } J_9=\begin{bmatrix} -(\mu_F+u_F)-\frac{\beta\xi(M^{**}+I^{**})}{N} & 0 & 0 \\ \frac{\beta\xi(M^{**}+I^{**})}{N} & -(\gamma+\mu_F+u_F) & 0 \\ 0 & \gamma & -(\mu_F+u_F) \end{bmatrix}.$$

Lemma 2 *The condition $a_n>0$ is necessary and sufficient for the epidemic equilibrium point P_1 to be locally asymptotically stable*[4].

Proposition 2 ***(The local stability of the epidemic equilibrium point).*** *The epidemic equilibrium point P_1 is an asymptotically stable node if $R_0>1$.*

Demonstration:

For the epidemic equilibrium point, applying P_1 to a_n and making $F^{**}=F_S^{**}+F_E^{**}+F_I^{**}$ we found:

$$a_n=\frac{\beta\,\xi\,F_S^{**}\,\varepsilon\,\phi\,\mu\left(\frac{\xi\,\psi F_I^{**}}{N}+\mu\right)(\nu+\mu)(\theta_M+\mu)(\theta_I+\mu)\left(\frac{\varepsilon\phi F^{**}}{C}+\alpha+\mu_A+u_A\right)\left(\mu_F+u_F+\frac{\beta\xi(M^{**}+I^{**})}{N}\right)(\gamma+\mu_F+u_F)(\mu_F+u_F)(A^{**}-C)}{CN}$$

Thus, the epidemic equilibrium point P_1 is locally asymptotically stable for $R_0>1$.

□

Variation of the model's parameters

To exemplify the variation that some of the model's parameters suffer due to the climatic variables of precipitation, temperature, and humidity, consider Figure S2.

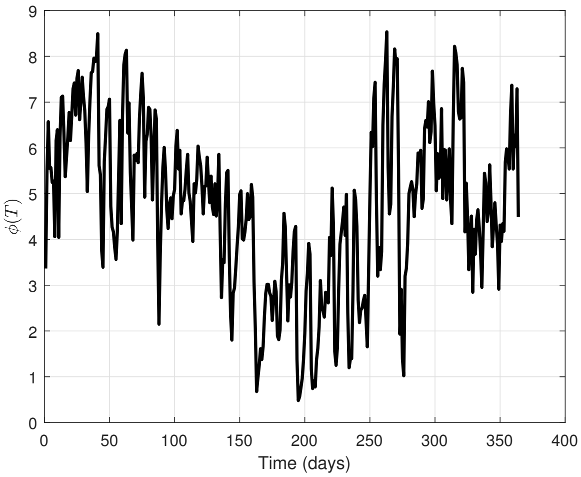

a Oviposition rate by each female

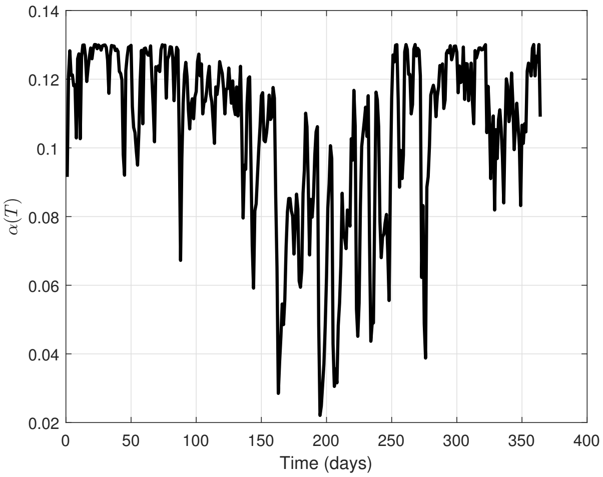

b Development rate of immatures to adults

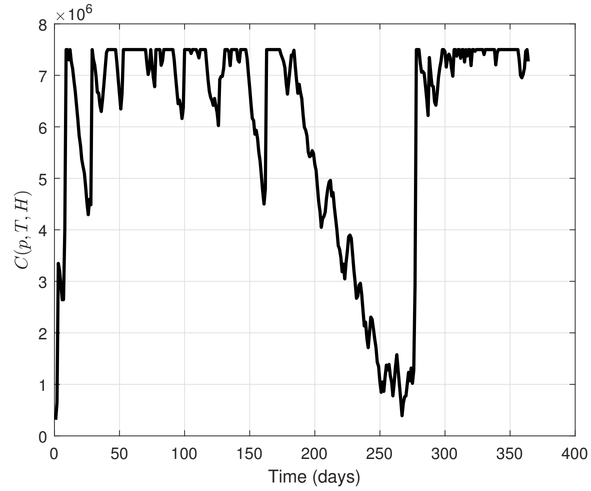

c Carrying capacity

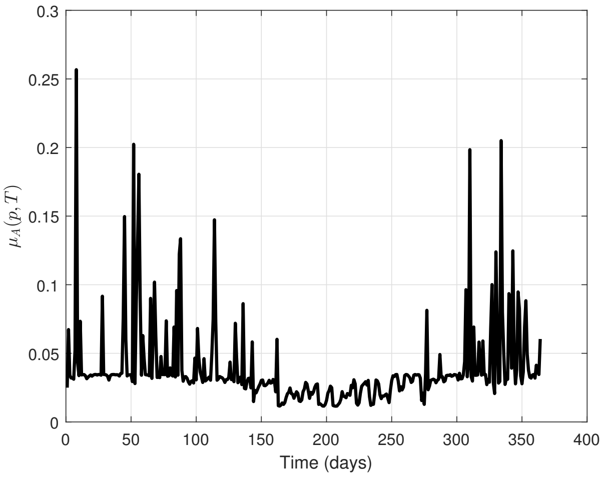

d Natural death rate of eggs, larvae and pupae

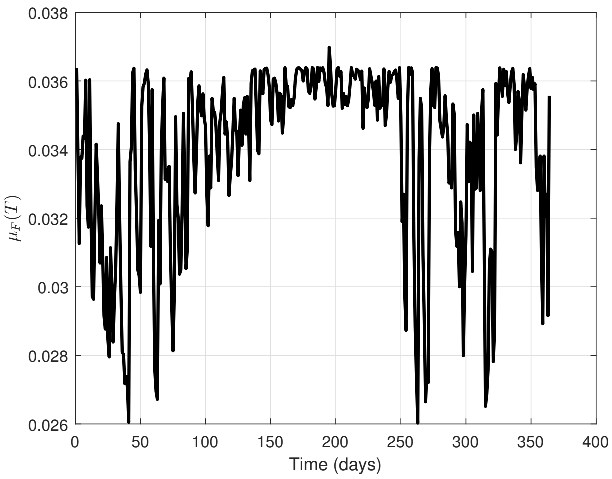

e Natural death rate of female mosquitoes

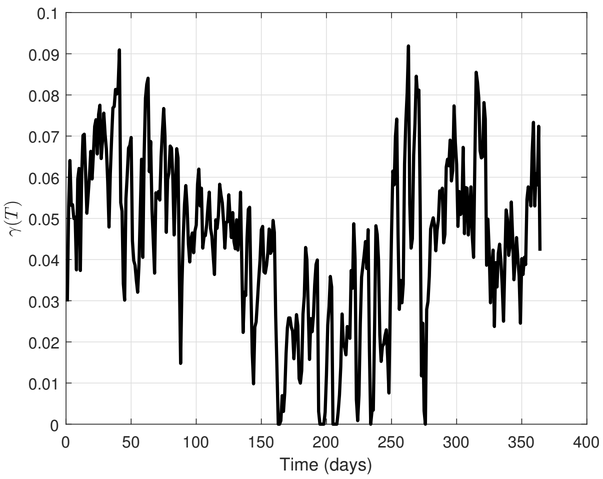

f Extrinsic incubation rate of mosquitoes

Figure S2. Evolution of each model parameter that is influenced by precipitation, temperature, and humidity.

Results

Decision spaces

Figure S3 and Figure S4 show the decision spaces of the five cycles of larvicidal and perifocal control, respectively, while Figure S5 shows the decision space of the two ULV spraying cycles. About the results presented in the figures, some remarks are required. Most of the time, the entire search space for the control decision variable was covered, in contrast to the time decision variable, which was more concentrated in some specific regions. As expected, because of the stochastic nature of a real problem, there was no standard behavior in the distribution of points in the decision spaces. Moreover, even when dealing with the same decision variable, the behavior was different in each control cycle. For the larvicide control (u_A), the highest concentration of points was at the lower bound of up to 1% of additional control per cycle.

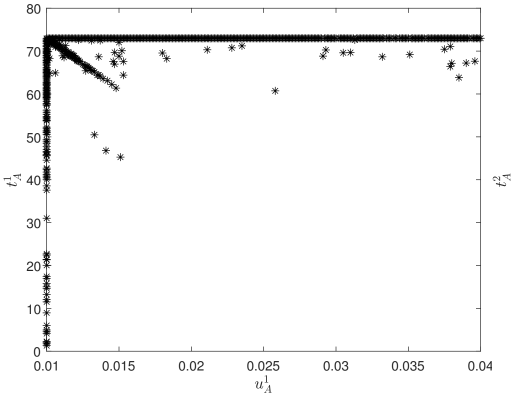

a The 1st u_A cycle.

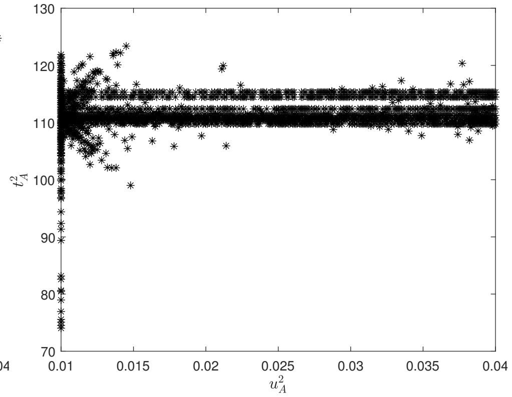

b The 2nd u_A cycle.

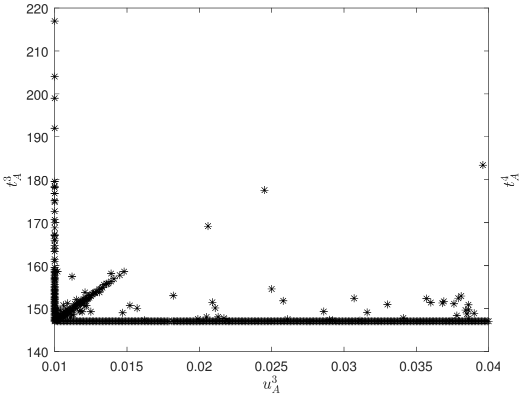

c The 3rd u_A cycle.

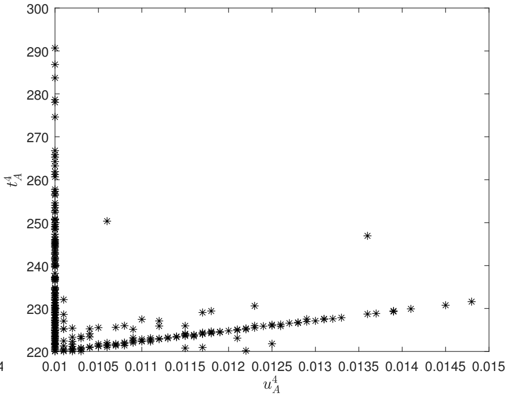

d The 4th u_A cycle.

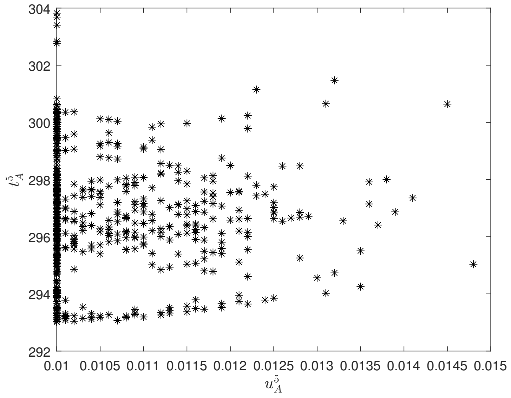

e The 5th u_A cycle.

Figure S3. Larvicide control decision variable space.

As for the perifocal control (u_{F_1}), the highest concentration of points was at the upper bound of up to 2% of additional control per cycle.

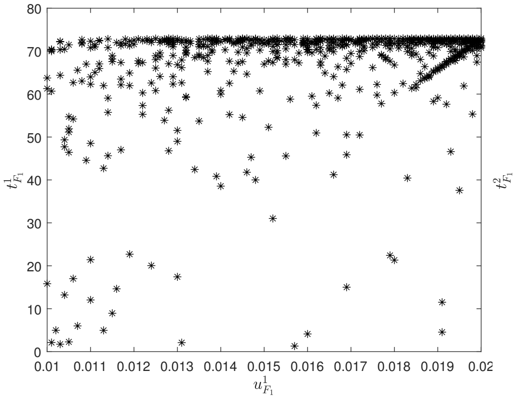

a The 1st u_{F_1} cycle.

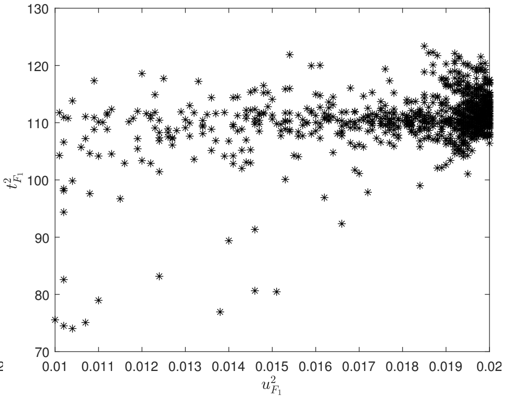

b The 2nd u_{F_1} cycle.

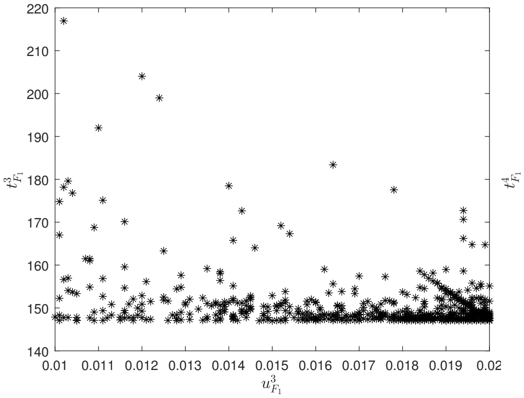

c The 3rd u_{F_1} cycle.

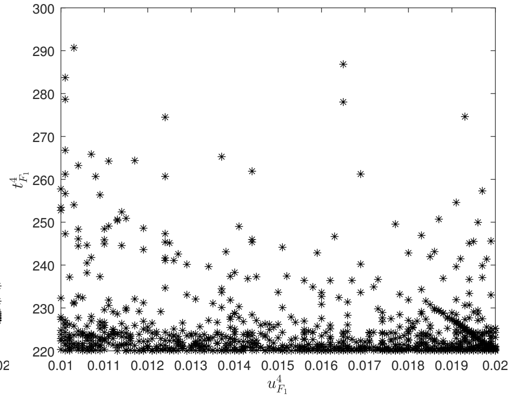

d The 4th u_{F_1} cycle.

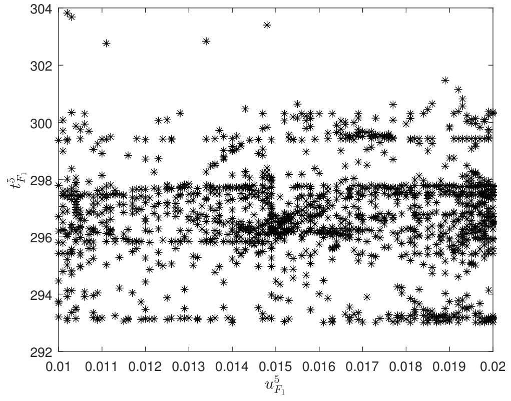

e The 5th u_{F_1} cycle.

Figure S4. Perifocal control decision variable space.

Finally, for the ULV treatment (u_{F_2}), the highest concentration of points was at the lower bound of up to 1% of additional control per cycle.

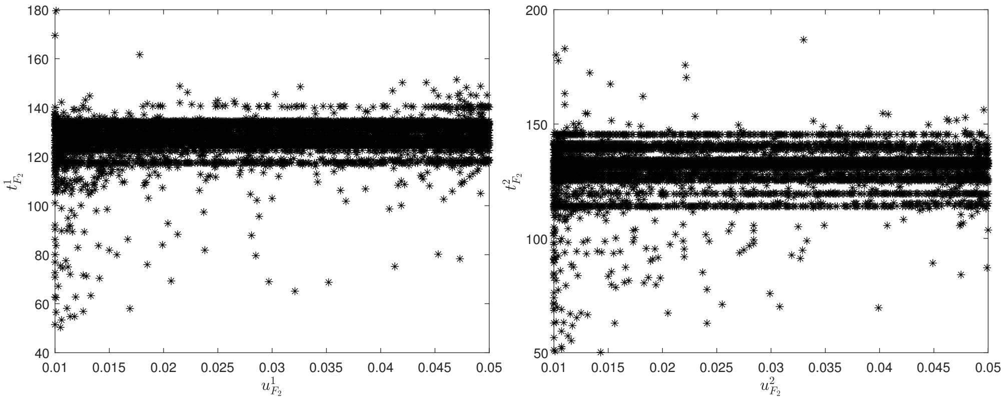

a The 1st u_{F_2} cycle. **b** The 2nd u_{F_2} cycle.

Figure S5. Ultra-low volume spraying decision variable space.

Pareto fronts

Figure S6a intends to display the combined result of all Pareto fronts found in the objective space, that is, the nondominated front. This result includes the 30 runs of the NSGA-II before excluding nondominated points. Therefore, the border is formed by 15,000 points. We can see a greater concentration of points in the region that corresponds to the balance between the two objective functions, which the literature calls the "knee" of a curve. Figure S6b shows the final Pareto front. It is composed of 840 combined nondominated points found in all algorithmic runs.

Figure S6b shows that the Pareto front has three well-defined regions. First, an almost horizontal region, close to the J_1 function axis; second, an almost vertical region, close to the J_2 function axis; and a third proportional region between the two previous regions, which corresponds to the knee.

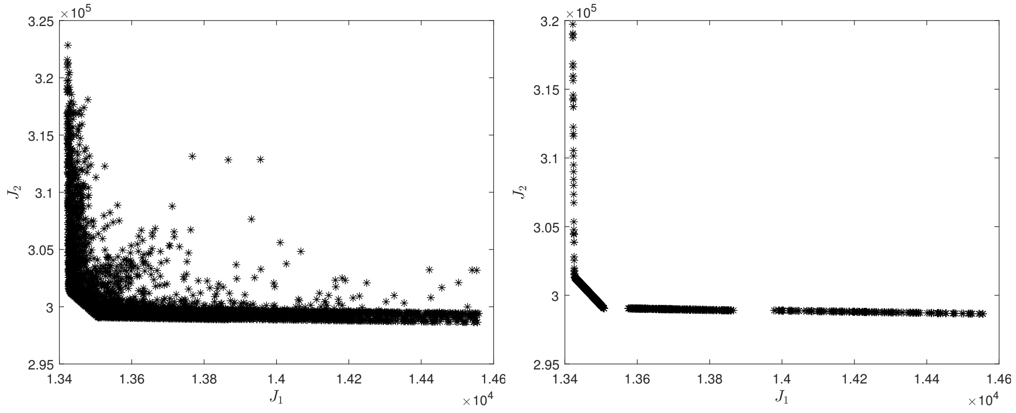

a Nondominated front. **b** Combined nondominated front.

Figure S6. Nondominated front in the objectives space J_1 (the control costs) $\times J_2$ (the hospital costs).

References

1. Van den Driessche, P. & Watmough, J. Reproduction numbers and sub-threshold endemic equilibria for compartmental models of disease transmission. *Math. Biosci.* **180**, 29–48, DOI: https://doi.org/10.1016/S0025-5564(02)00108-6 (2002).
2. Heesterbeek, J. A. P. A brief history of R_0 and a recipe for its calculation. *Acta biotheoretica* **50**, 189–204, DOI: https://doi.org/10.1023/a:1016599411804 (2002).
3. Forattini, O. *Família Culicidae Biologia das Formas Imaturas; Biologia Geral das Formas Adultas*, vol. 2, chap. 2–3, 51–115 (EDUSP, São Paulo, SP, 1996).
4. Leite, M. B. F., Bassanezi, R. C. & Yang, H. M. The basic reproduction ratio for a model of directly transmitted infections considering the virus charge and the immunological response. *Math. Medicine Biol. a J. IMA* **17**, 15–31 (2000). https://pubmed.ncbi.nlm.nih.gov/10757030.
